# GATA4 induces liver fibrosis regression by deactivating hepatic stellate cells

**DOI:** 10.1172/jci.insight.150059

**Published:** 2021-12-08

**Authors:** Noelia Arroyo, Laura Villamayor, Irene Díaz, Rita Carmona, Mireia Ramos-Rodríguez, Ramón Muñoz-Chápuli, Lorenzo Pasquali, Miguel G. Toscano, Franz Martín, David A. Cano, Anabel Rojas

**Affiliations:** 1 Centro Andaluz de Biología Molecular y Medicina Regenerativa (CABIMER), Universidad Pablo de Olavide, Universidad de Sevilla, Consejo Superior de Investigaciones Científicas (CSIC), Seville, Spain.; 2 Centro de Investigación Biomédica en Red de Diabetes y Enfermedades Metabólicas Asociadas, Madrid, Spain.; 3 Universidad de Málaga y Centro Andaluz de Nanomedicina, Málaga, Spain.; 4 Department of Human Anatomy and Embryology, Legal Medicine and History of Medicine, Faculty of Medicine, University of Málaga, Málaga, Spain.; 5 Endocrine Regulatory Genomics, Department of Experimental & Health Sciences, University Pompeu Fabra, Barcelona, Spain.; 6 Amarna Therapeutics, Sevilla, Spain.; 7 Instituto de Biomedicina de Sevilla, Hospital Universitario Virgen del Rocío/CSIC/Universidad de Sevilla, Seville, Spain.

**Keywords:** Cell Biology, Gastroenterology, Fibrosis, Mouse models

## Abstract

In response to liver injury, hepatic stellate cells activate and acquire proliferative and contractile features. The regression of liver fibrosis appears to involve the clearance of activated hepatic stellate cells, either by apoptosis or by reversion toward a quiescent-like state, a process called deactivation. Thus, deactivation of active hepatic stellate cells has emerged as a novel and promising therapeutic approach for liver fibrosis. However, our knowledge of the master regulators involved in the deactivation and/or activation of fibrotic hepatic stellate cells is still limited. The transcription factor GATA4 has been previously shown to play an important role in embryonic hepatic stellate cell quiescence. In this work, we show that lack of GATA4 in adult mice caused hepatic stellate cell activation and, consequently, liver fibrosis. During regression of liver fibrosis, *Gata4* was reexpressed in deactivated hepatic stellate cells. Overexpression of *Gata4* in hepatic stellate cells promoted liver fibrosis regression in CCl_4_-treated mice. GATA4 induced changes in the expression of fibrogenic and antifibrogenic genes, promoting hepatic stellate cell deactivation. Finally, we show that GATA4 directly repressed *EPAS1* transcription in hepatic stellate cells and that stabilization of the HIF2α protein in hepatic stellate cells leads to liver fibrosis.

## Graphical abstract

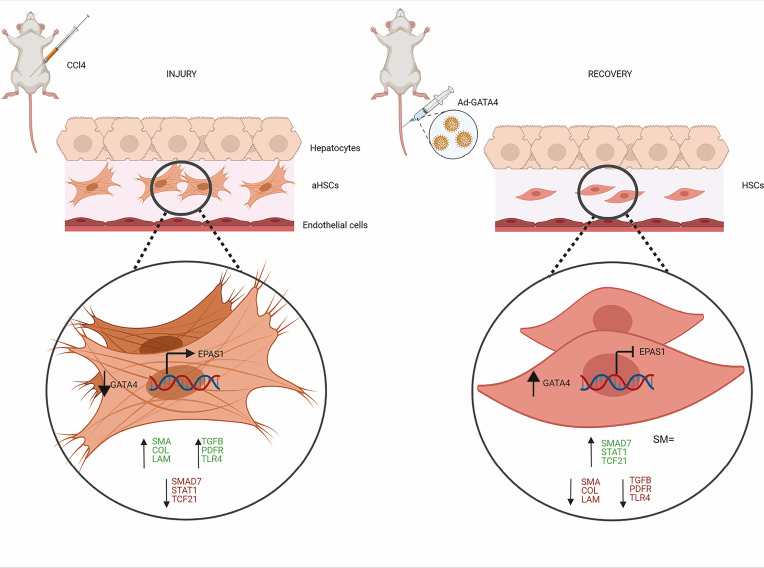

## Introduction

Liver fibrosis is characterized by the excessive accumulation of extracellular matrix (ECM) components that might eventually lead to cirrhosis and impaired hepatic function. Liver fibrosis is caused by different chronic liver pathologies, including hepatitis B and C virus infections, alcohol abuse, and nonalcoholic steatohepatitis (1). Regardless of the etiology, hepatic stellate cells (HSCs) are key players in the development of liver fibrosis. In response to liver injury, HSCs get activated, acquiring proliferative and contractile features and become myofibroblast-like cells that express α-smooth muscle actin (α-SMA) and synthesize ECM components such as collagen and laminin (2). The initial activation of HSCs is caused by paracrine signals from other liver cells, such as macrophages, hepatocytes, and endothelial cells (3). However, the maintenance of the active state of HSCs requires autocrine as well as paracrine loops (4). Among the signaling molecules shown to induce activation of HSCs are PDGF-β, TGF-β, and inflammatory cytokines (5). It has become increasingly clear in recent years that liver fibrosis can be reversed upon cessation of injury. The regression of liver fibrosis seems to involve the clearance of activated HSCs (aHSCs; refs. 6, 7). Lineage-tracing studies in mouse models have demonstrated that, during the regression of liver fibrosis, approximately half of the aHSCs escape from apoptosis or cell senescence and revert their phenotype to an inactive or quiescent-like state, a process called deactivation (8). This deactivation phenomenon has also been recently described in human HSCs (9). In this context, targeting the deactivation of aHSCs has emerged as a novel and promising therapeutic approach for liver fibrosis.

However, our knowledge of the underlying molecular mechanisms, in particular master regulators, involved in aHSC deactivation is still limited. We have previously shown the zinc finger type transcription factor GATA4 plays a critical role in HSC phenotype during embryonic development. Inactivation of *Gata4* specifically in HSCs results in aberrant accumulation of ECM components and activation of HSCs in embryonic livers (10). Here, we aimed to determine the role of GATA4 in HSC deactivation during adult liver fibrosis regression. We show that GATA4 induced the deactivation of adult aHSCs. We also demonstrate that GATA4 regulated the expression of the endothelial PAS domain-containing protein 1 gene (*EPAS1*) and that EPAS1/HIF2α activity in HSCs induces liver fibrosis in mice.

## Results

### GATA4 prevents adult HSC activation.

Our previous work demonstrated that *Gata4* inactivation in embryonic HSCs, using the G2-Cre line, leads to liver fibrosis, and therefore, this transcription factor is required to maintain the quiescence of embryonic HSCs (10). The G2-Cre line directs the Cre recombinase in the septum transversum and its derivatives, including HSCs and epicardial cells. The embryonic lethality of *G2-Cre*; *Gata4^fl/fl^* mice precludes the study of GATA4 function in adult HSCs. To determine whether GATA4 regulates adult HSC quiescence in mice, we used an adenovirus-mediated system to deliver the Cre recombinase. *Gata4^fl/fl^* adult mice were injected with Cre-expressing adenoviruses (Ad-Cre) via the tail vein. GFP-expressing adenoviruses (Ad-GFP) were used as control. Mice were sacrificed for analysis 2 weeks after adenovirus injections. Previous studies have shown that adenovirus injection in the tail vein infects mostly and robustly liver cells, including hepatocytes, Kupffer cells, and HSCs (11). Indeed, quantification of GFP/desmin-positive cells in the livers of mice injected with 4.6 × 10^8^ viral genome of Ad-GFP revealed an efficient targeting of adenoviruses in HSCs. Around 51% of HSCs were transduced with Ad-GFP, whereas 14% were observed in hepatocytes and 11% in endothelial cells (Supplemental Figure 1; supplemental material available online with this article; https://doi.org/10.1172/jci.insight.150059DS1).

**Figure 1:**
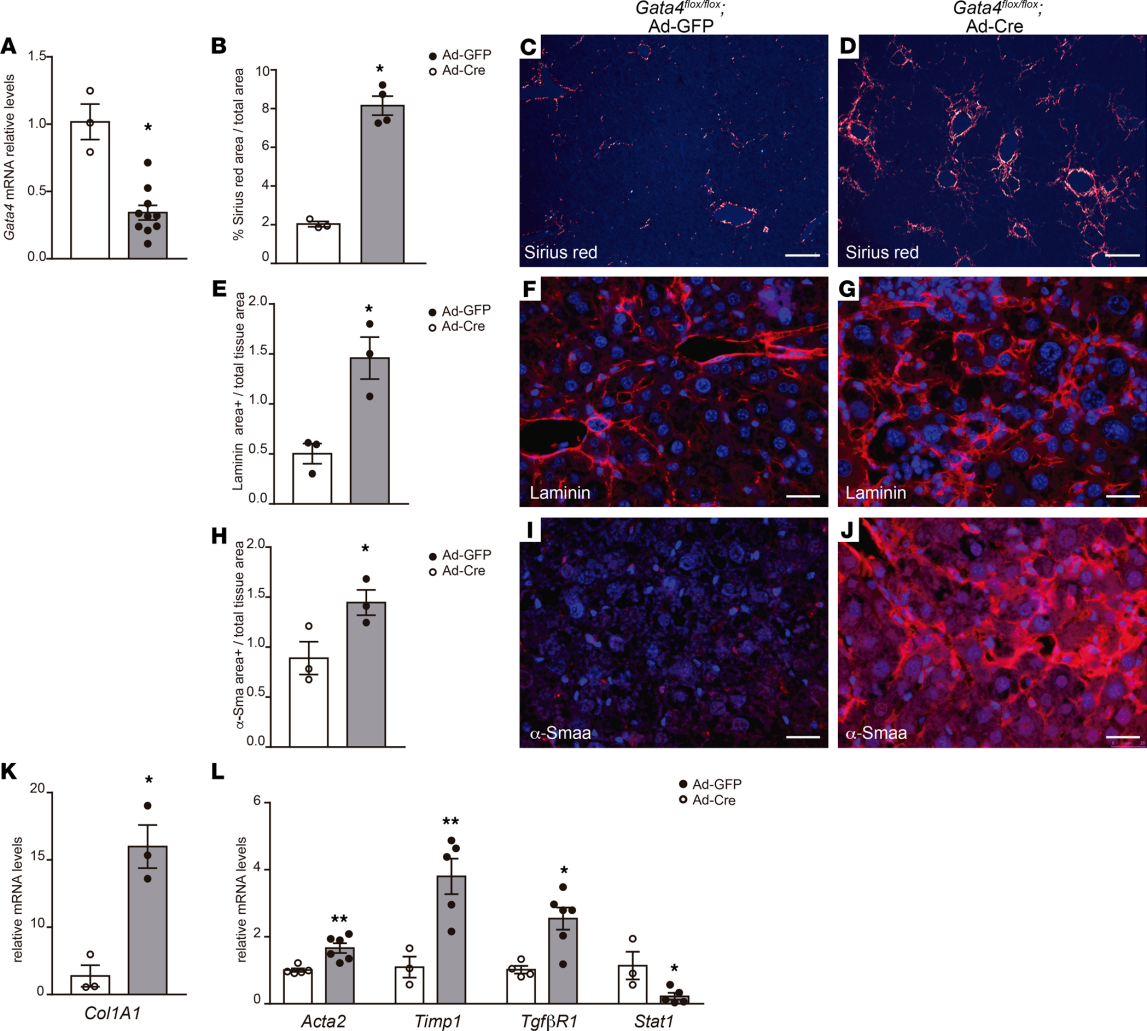
*Gata4* inactivation induces liver fibrosis in adult stages. (**A**) Quantitative RT-PCR analysis of *Gata4* expression in livers of adult *Gata4*-floxed mice injected with GFP-expressing (Ad-GFP) or *Cre*-expressing adenoviruses (Ad-Cre) (Ad-GFP *n =* 3; Ad-Cre = 10). (**B**) Quantification of Sirius red–stained area per total liver area in Ad-GFP– and Ad-Cre–injected mice (*n =* 3, Ad-GFP; *n =* 4, Ad-Cre). Polarized light microscopy images of Sirius red–stained liver sections from *Gata4-*floxed mice treated with (**C**) Ad-GFP and (**D**) Ad-Cre adenoviruses. (**E**) Quantification of the liver area immunostained for the ECM protein laminin in Ad-GFP– and Ad-Cre–injected mice (*n =* 3 each group). Increased accumulation of the ECM protein laminin in the liver of (**F**) Ad-GFP– and (**G**) Ad-Cre–injected mice (*n =* 3, Ad-GFP; *n* = 4, Ad-Cre). (**H**) Quantification of the liver area immunostained for α-smooth muscle actin (SMA) in Ad-GFP– and Ad-Cre–injected mice (*n =* 3 each group). Activation of HSCs, marked by α-smooth muscle actin is observed in (**J**) *Gata4*-floxed mice treated with Ad-Cre adenoviruses compared with (**I**) *Gata4*-floxed mice treated with Ad-GFP adenoviruses. Quantitative RT-PCR analysis of (**K**) *Col1A1* and (**L**) *Acta2*, *Timp1*, *Tgf*β*R1*, and *Stat1* expression in livers of Ad-GFP– and Ad-Cre–injected mice (*n =* 3–5, Ad-GFP; *n* = 5–6, Ad-Cre). Scale bars: 100 μm (**C** and **D**); 25 μm (**F**, **G**, **I**, and **J**). Statistical analyses was performed using 2-tailed Student’s test. Error bars represent mean ± SEM. *P < 0.05, **P < 0.01.

*Gata4*-floxed mice treated with Ad-Cre displayed a marked decrease in *Gata4* mRNA levels in liver, indicating an efficient inactivation of the *Gata4*-floxed allele by the Cre recombinase (Figure 1A). Efficient excision of the *Gata4*-floxed allele was further confirmed by PCR of genomic liver DNA (Supplemental Figure 1). Of note, GATA4 accumulation at the protein level could not be properly analyzed in *Gata4*-floxed mice. Additionally, the identification of the truncated version of the GATA4 protein (which is encoded by this particular floxed allele; ref. 12) with commercial antibodies was not reliable. Inactivation of *Gata4* led to an increase in collagen (stained with Sirius red) and laminin accumulation (Figure 1, B, D, E, and G) in the livers of Ad-Cre–treated mice compared with *Gata4*-floxed mice injected with Ad-GFP (Figure 1, B, C, E, and F). The accumulation of ECM in *Gata4*-floxed mice injected with Ad-Cre was concomitant with HSC activation, as demonstrated by the prominent expression of α*-Sma* (Figure 1, H and J), compared with mice treated with Ad-GFP (Figure 1, H and I). Quantitative RT-PCR analysis confirmed the increase in the expression of fibrotic markers, including collagen 1A1 (Col1A1), *Acta2*, *Timp1*, and *Tgf*β*R1* (Figure 1, K and L). Conversely, the expression levels of STAT1, a negative regulator of liver fibrosis, were reduced (Figure 1L). Of note, injection of Ad-GFP and Ad-Cre in C56BL/6 wild-type mice did not cause any defects in liver histology (Supplemental Figure 1). Altogether, these data indicate that GATA4 is required to maintain the quiescent state of adult HSCs.

### Gata4 is reexpressed in HSCs during regression of liver fibrosis.

**Figure 2:**
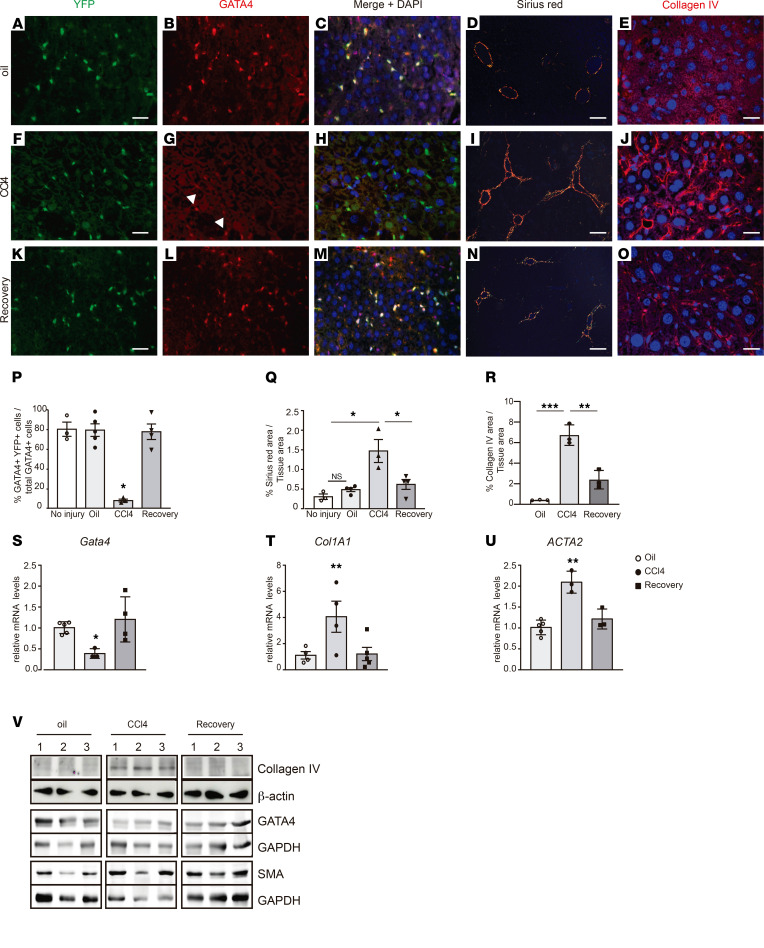
Lineage tracing of *G2-Cre*–derived cells and *Gata4* expression during adult liver fibrosis and recovery. (**A**–**O**) Liver sections of *G2-Cre;Rosa26ReYFP* adult mice. Immunostaining for (**A**, **F**, and **K**) YFP and (**B**, **G**, and **L**) GATA4 in liver sections of mice treated with vehicle (oil) (**A** and **B**) and CCl_4_ for 4 weeks (**F** and **G**) and 1 month after CCL_4_ treatment (recovery phase) (**K** and **L**). (**C**, **H**, and **M**) Merged images of YFP, GATA4, and DAPI staining. Polarized light microscopy images of Sirius red–stained liver sections of (**D**) mice treated with oil, (**I**) mice treated with CCl_4_, and (**N**) mice 1 month after recovery. (**A**–**L**, *n =* 3 each group). Immunostaining for collagen IV in liver sections of (**E**) mice treated with oil, (**J**) mice treated with CCl_4_, and (**O**) mice 1 month after recovery. (**P**) Quantification of double GATA4/YFP-positive cells per total GATA-positive cells in livers of each experimental group (*n =* 3–5 each group). (**Q**) Relative quantification of Sirius red–stained area per total liver area in each experimental group (*n =* 3–4 each group). (**R**) Relative quantification of collagen IV–stained area per total liver area in each experimental group (*n =* 3 each group). The no injury group denotes mice not injected with adenovirus or CCL4. Quantitative RT-PCR analysis of (**S**) *Gata4*, (**T**) *Col1A1*, and (**U**) α*-Sma* expression in each experimental group (*n =* 3–5 each group). (**V**) Western blot analysis of GATA4, collagen IV, and α-SMA accumulation in livers of mice treated with oil, mice treated with CCl_4_, and mice 1 month after recovery. β-Actin protein or GAPDH was used for loading control. Samples from 3 independent mice in each experimental group are shown. Scale bars: 25 μm (**A**–**C**, **F**–**H**, **K**–**M**, **E**, **J**, and **O**); 100 μm (**D**, **I**, and **N**). Statistical analyses was performed using 1-way ANOVA. Error bars represent mean ± SEM.*P < 0.05. **P < 0.01.

Next, we decided to analyze the *Gata4* expression pattern in adult HSCs during the development of liver fibrosis and posterior regression in the CCl_4_-induced liver injury model. To determine whether *Gata4* is reexpressed in HSCs during the regression of liver fibrosis, we performed lineage-tracing analysis by crossing the HSC-specific *G2-Cre* mouse line with *ROSA26R-eYFP* mice. At adult stages, the majority of HSCs, as determined by desmin accumulation, expressed YFP (76%; Supplemental Figure 2). A similar percentage of YFP-labeled cells were positive for GATA4 (Figure 2, A–C and P). As expected, mice treated with CCl_4_ show increased accumulation of collagen fibers in the liver compared with mice treated with vehicle (Figure 2, D, I, and Q). Immunostaining for collagen IV confirmed accumulation of this ECM protein in CCl4-treated mice compared with oil-treated mice (Figure 2, E and J). Several reports have found that female rodents are more resistant to CCl_4_ treatment than male rodents (13, 14). However, we did not find different susceptibility toward CCl_4_ between male and female mice, as assessed by liver collagen fiber accumulation or serum ALT and AST levels (Supplemental Figure 3), likely due to the low levels of CCl_4_ used in this experiment. In agreement with the elevated accumulation of collagen fibers observed in CCl_4_-treated mice, the expression of Col1A1 and the activation marker for HSCs, α*-Sma*, was increased (Figure 2, I, T, and U). A dramatic downregulation of *Gata4* expression in HSCs from CCl_4_-treated mice was observed (Figure 2, F–H, P, S, and V; see complete unedited blots in the supplemental material). After the recovery period (1 month after CCl_4_ treatment), collagen fibers in CCl_4_-treated mouse livers, including collagen IV, were clearly diminished, and downregulation of *Col1A1* and α*-Sma* expression and protein levels was observed (Figure 2, N, O, Q, R, and T–V). Remarkably, the reduced expression of *Gata4* was fully restored in YFP-labeled deactivated HSCs after the recovery period (Figure 2, K–M, P, S, and V). Analysis of *Gata4* expression levels at different time points after the cessation of CCl_4_ treatment (2, 3, and 4 weeks) revealed a full reactivation of endogenous GATA4 expression in 2 weeks (Supplemental Figure 4). However, clearance of collagen accumulation was only observed after 4 weeks of recovery (Supplemental Figure 4). These results indicate that *Gata4* is reexpressed in aHSCs that return to a quiescent state during the regression of liver fibrosis.

### Gata4 overexpression reduces liver fibrosis in vivo.

**Figure 3:**
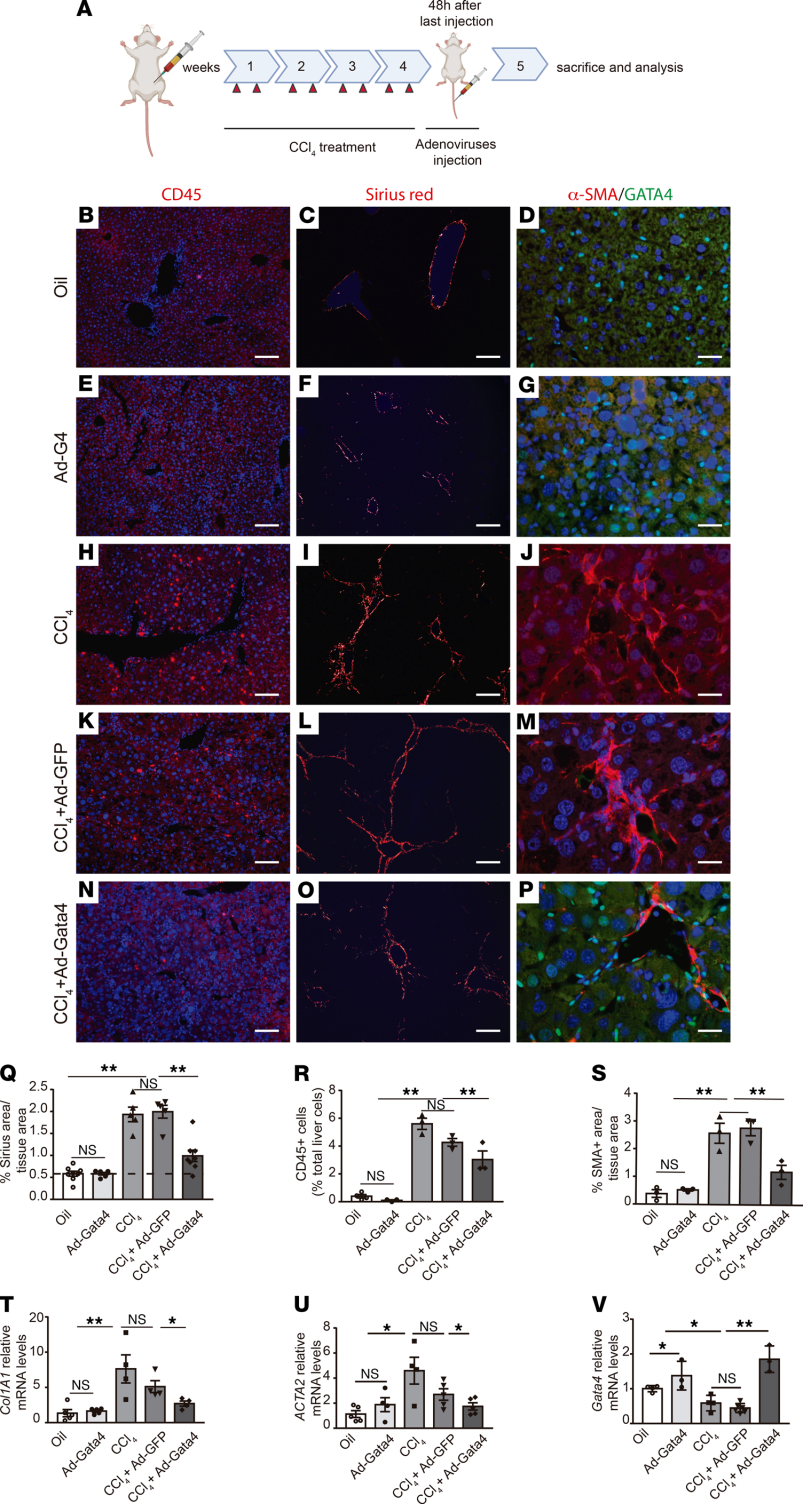
GATA4 promotes the regression of liver fibrosis regression. (**A**) Schematic of the experimental design. To induce liver fibrosis, CCl_4_ was administered to adult wild-type C57BL/6 mice for 4 weeks. Two days after the last CCl_4_ injection, mice were infected with GFP-expressing (Ad-GFP) and *Gata4*-overexpressing (Ad-Gata4) adenoviruses via the tail vein. Liver tissue is analyzed 1 week after adenovirus infection. Wild-type C57BL/6 mice treated with CCL_4_ showed an increase in inflammatory cells, (**H**) marked by CD45 immunostaining, (**I**) accumulation of collagen fibers marked with Sirius red staining, and (**J**) activation of HSCs, marked by α-SMA accumulation, compared with control mice treated with vehicle (oil) (**B**, **C**, and **D**, respectively). Infection of Ad-GATA4 adenoviruses in wild-type untreated C57BL/6 mice did not effect (**E**) CD45 cell numbers, (**F**) collagen accumulation, and (**G**) α-SMA accumulation. Increased numbers of CD45-positive cells, α-SMA expression in HSCs and collagen accumulation were observed in CCl_4_-treated mice followed by administration of Ad-GFP adenoviruses (**K**–**M** and **Q**–**U**). A remarkable decrease in (**N**) the number of inflammatory cells, (**O**) collagen accumulation, and (**P**) HSC activation was observed after administration of Ad-GATA4 adenoviruses to CCl_4_-treated mice compared with CCl_4_-treated mice infected with Ad-GFP adenoviruses (**K**, **L**, and **M**, respectively). (**Q**) Relative quantification of Sirius red–stained area per total liver area in each experimental group (*n =* 5–8). (**R**) Quantification of CD45-positive cells per total liver cells in each experimental group (*n =* 3 each group). (**S**) Relative quantification of α-SMA–positive area per total liver area in each experimental group (*n =* 3 each group). Quantitative RT-PCR analysis of (**T**) *Col1A1* (*n =* 4–5 each group), (**U**) α*-Sma* (*n =* 4–5 each group), and (**V**) *Gata4* (*n =* 3–5 each group) expression in each experimental group. Scale bars: 100 μm (**B**, **C**, **E**, **F**, **H**, **I**, **K**, **L**, **N**, and **O**); 25 μm (**D**, **G**, **J**, **M**, and **P**). Statistical analyses was performed using 1-way ANOVA. Error bars represent mean ± SEM. *P < 0.05, **P < 0.01.

The results described above suggest a role of GATA4 in the deactivation of adult HSCs during liver fibrosis regression. To test this hypothesis, we evaluated the effect of *Gata4* overexpression on liver fibrosis regression in vivo. To this end, mice were treated with CCl_4_ for 4 weeks to induce liver fibrosis, followed by tail vein injection of *Gata4*-overexpressing adenovirus (Ad-GATA4) or Ad-GFP as a control. Liver tissues were collected 1 week after adenovirus infection, i.e., 9 days after the last CCl_4_ injection (Figure 3A). Histological evaluation using Sirius red revealed diminished fibrillar collagen deposition in the livers of mice injected with Ad-GATA4 compared with both control mice (no infection with adenoviruses) and mice injected with Ad-GFP (Figure 3, C, I, L, O, and Q). Decreased CD45 immunostaining indicated reduced liver inflammation in mice injected with Ad-GATA4 (Figure 3, B, H, K, N, and R). Of note, injection of Ad-GATA4 in wild-type mice did not have any effect on collagen accumulation or liver inflammation (Figure 3, E–G, Q, and R). We evaluated HSC activation in CCl_4_-treated mice by α-SMA immunohistochemistry. Livers of CCl_4_-treated mice injected with Ad-GATA4 showed a decreased number of α-SMA–positive areas compared with both control mice (no infection with adenoviruses) and mice injected with Ad-GFP (Figure 3, D, J, M, P, and S). Quantification of *Col1A1* and α*-Sma* mRNA levels confirmed a reduction of fibrosis and HSC activation in *Gata4*-overexpressing mice (Figure 3, T–V). Thus, *Gata4* overexpression promotes liver fibrosis regression in vivo by deactivating HSCs.

### GATA4 reverts the active phenotype of HSCs by modulating the expression of fibrogenic and antifibrogenic genes.

**Figure 4:**
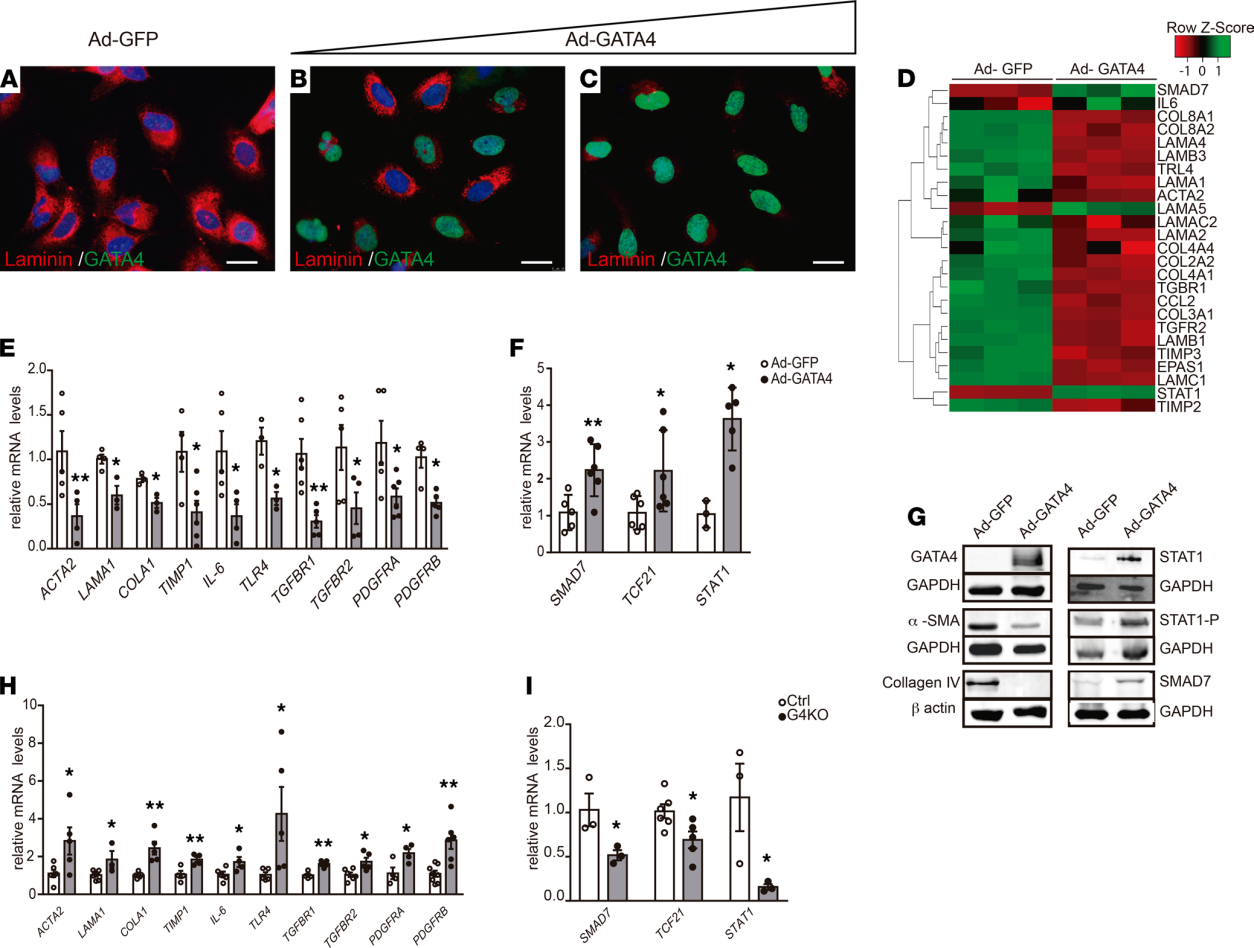
GATA4 regulates the expression of fibrogenic and antifibrogenic genes in HSCs. Immunofluorescence analyses of laminin and GATA4 accumulation in LX2 cells transfected with (**A**) GFP-expressing (Ad-GFP) (multiplicity of infection [MOI], = 100) and GATA4-expressing (Ad-GATA4) adenovirus at (**B**) MOI = 50 and (**C**) MOI = 100. (**D**) Heatmap showing the most differentially expressed genes in Ad-GATA4–transfected LX2 cells compared with LX2 cells transfected with Ad-GFP. Validation by quantitative RT-PCR analysis of differentially (**E**) fibrogenic (*n =* 3–6) and (**F**) antifibrogenic (*n =* 3–6) expressed genes (Ad-GATA4 vs. Ad-GFP infected LX2 cells) identified in the microarray analyses. (**G**) Validation by Western blot analysis of selected differentially expressed genes (Ad-GATA4 vs. Ad-GFP infected LX2 cells) identified in the microarray analyses. β-Actin and GAPDH proteins were used as loading controls. Quantitative RT-PCR analysis of (**H**) fibrogenic (*n =* 4–8) and (**I**) antifibrogenic genes (*n =* 3–6) in *G2-Cre;Gata4 KO* E13.5 embryonic liver. Scale bars: 25 μm. Statistical analyses was performed using 2-tailed Student’s test. Error bars represent mean ± SEM. *P < 0.05, **P < 0.01.

To confirm the cell-autonomous role of GATA4 in HSC deactivation and to get insight into the underlying molecular mechanisms, we turned to cell culture using LX2 cells, a human HSC cell line that recapitulates many features of the aHSC phenotype. We devised a system that allows robust *Gata4* activation in LX2 cells using Ad-GATA4 or Ad-GFP as a control. Adenovirus-mediated overexpression of *Gata4* caused a marked reduction in laminin immunoreactivity, which further decreased as a multiplicity of infection (MOI) increased (Figure 4, B and C). At a MOI of 100, nearly 98% of LX2 cells were transduced. Transduction of LX2 with Ad-GFP at a MOI of 100 did not affect laminin expression (Figure 4A). Next, microarray analyses were performed to analyze changes in gene expression in *Gata4*-overexpressing LX2 cells compared with GFP-overexpressing LX2 cells (Figure 4D). Among the most downregulated genes were profibrogenic genes such as ECM components (*ACTA2*, *LAMA1*, and *COLlA1*), metalloprotease inhibitors (*TIMP1*), TGF-β receptors (*TGF*β*R1* and *TGF*β*R2*) and PDGF receptors (*PDGFRA* and *PDGRFB*), *TLR4*, and *IL6*. Conversely, an increase in the expression of known antifibrogenic factors, such as *STAT1*, *SMAD7*, and the transcription factor *TCF21*, was observed. Quantitative RT-PCR and Western blot analysis confirmed the changes in expression in these fibrosis-related genes (Figure 4, E–G). Interestingly, these genes showed the reverse expression pattern in the liver tissue of embryos lacking *Gata4* specifically in HSCs (10), confirming that GATA4 regulates the expression of multiple genes involved in liver fibrosis (Figure 4, H and I).

### EPAS1 is a direct target of GATA4.

**Figure 5:**
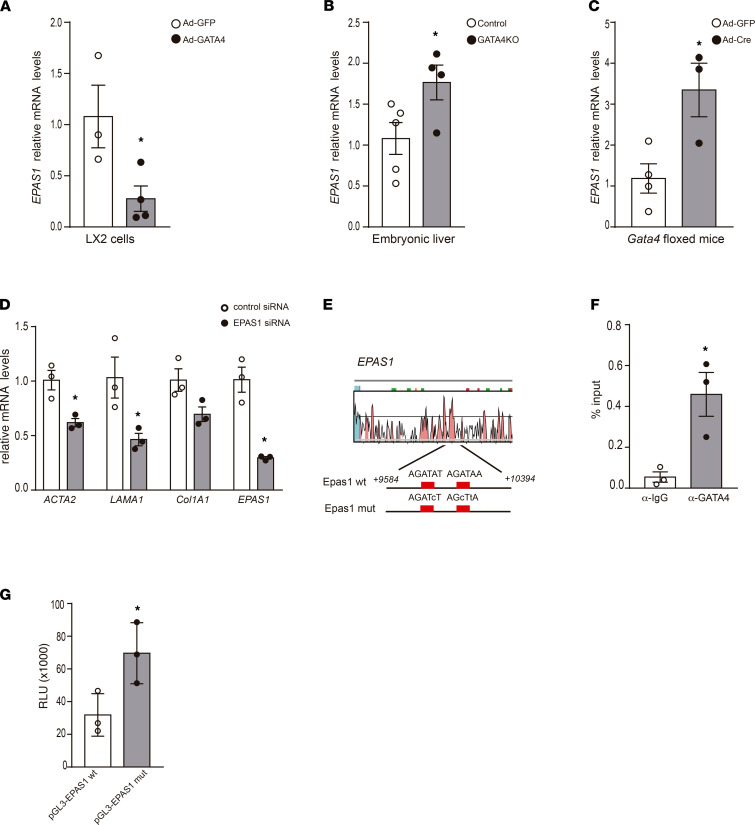
GATA4 is a direct repressor of *EPAS1* in HSCs. (**A**) Quantitative RT-PCR analysis of *EPAS1* expression in LX2 cells transfected with GFP-expressing (Ad-GFP) and GATA4-expressing (Ad-GATA4) adenoviruses (*n* = 3, Ad-GFP; *n* = 4, Ad-GATA4). (**B**) Quantitative RT-PCR analysis of *EPAS1* expression in *G2-Cre;Gata4 KO* E13.5 embryonic liver (*n* = 5, control; *n* = 4, GATA4 KO). (**C**) Quantitative RT-PCR analysis of *EPAS1* expression in livers of adult *Gata4*-floxed mice injected with GFP-expressing (Ad-GFP) (*n =* 4) and *Cre*-expressing adenoviruses (Ad-Cre) (*n =* 3). (**D**) Quantitative RT-PCR analysis in LX2 cells transfected with a siRNA directed against *EPAS1* compared with control LX2 cells (treated with siRNA-negative control) (*n =* 3). (**E**) Schematic of the human *EPAS1* intronic region containing 2 conserved GATA sites using Vista Tools software. The blue peak indicates the human *EPAS1* transcription start, and the pink peaks indicate conserved noncoding regions between human and mouse. An 810 bp fragment of *EPAS1* intronic region containing the 2 conserved GATA4 sites (*EPAS1 wt*) or the mutated version (*EPAS1 mut*) was cloned into the pGL3 luciferase vector for reporter assays. The nucleotides mutated in *EPAS1 mut* are shown in lowercase. The numbers indicate the localization in the *EPAS1* locus from the transcriptional start site. (**F**) ChIP of LX2-overexpressing *GATA4* using a GATA4-specific antibody or a IgG-unspecific antibody (*n =* 3). (**G**) In vitro luciferase reporter assays in 293T cells of *pGL3-EPAS1 wt* and *pGL3-EPAS1 mut* plasmids (*n =* 3). Statistical analyses was performed using 2-tailed Student’s test. Error bars represent mean ± SEM. *P < 0.05. RLU, relative light units.

One of the most downregulated genes in the microarray data was EPAS1, encoding the hypoxia-inducible factor HIF2α. Quantitative RT-PCR analysis confirmed the decreased expression of *EPAS1* in *Gata4*-overexpressing LX2 cells (Figure 5A). Interestingly, *Epas1* expression was increased in the liver tissue of embryos lacking *Gata4* specifically in HSCs as well as in the livers of adult *Gata4*-floxed mice treated with Ad-Cre, further supporting an association between GATA4 and *Epas1* expression (Figure 5, B and C). To directly evaluate the role of HIF2α in HSC function, we performed knockdown experiments in LX2 cells using a specific siRNA against *EPAS1*. LX2 cells transfected with the *EPAS1* siRNA exhibited downregulation of the fibrotic markers *ACTA2*, collagen, and laminin (Figure 5D), revealing a role of HIF2α in the regulation of HSCs phenotype. Bioinformatic analyses of the *EPAS1* conserved regions identified 2 GATA4-binding sites within the first intron, localized at 9717 bp after the transcriptional start site (Figure 5E), suggesting that *EPAS1* was a direct target of GATA4. ChIP qPCR analysis validated these sites as GATA bona fide binding sites (Figure 5F). To examine the ability of GATA4 to repress the *Epas1* promoter, we generated reporter constructs containing the intronic region of *Epas1* with the wild-type version of the 2 GATA sites or the mutated version (Figure 5E) fused to the luciferase gene to generate the pGL3-*Epas1* wt and pGL3-*Epas1* *mut* reporter plasmids, respectively. We have previously reported endogenous expression of *Gata4* in 293T cells (15). Transient transfection of the pGL3-*Epas1 wt* reporter plasmid in 293T cells resulted in significantly lower luciferase activity compared with 293T cells transfected with pGL3-*Epas1mut* reporter plasmid (Figure 5G). Altogether, these results indicate that GATA4 directly represses *EPAS1* transcription through the 2 identified GATA sites.

### Stabilization of HIF2α induces liver fibrosis.

The results described above suggest that *EPAS1* expression was associated with HSC activation and liver fibrosis. We decided to evaluate the biological role of HIF2α in HSC function in vivo. To test this hypothesis in vivo, we specifically activated HIF2α in HSCs by crossing the HSC-specific *G2-Cre* mouse line with *HIF2dPA* mice (*G2-Cre;HIF2dPA* mice; ref. 16). *HIF2dPA* mice harbor a modified version of the human HIF2α that escapes recognition by the von Hippel-Lindau protein (i.e., it is not degraded under normal oxygen levels). Upon Cre recombination, excision of a floxed stop cassette allows the expression of the nondegradable form of HIF2α. *G2-Cre;HIF2dPA* mice displayed embryonic lethality between E15.5 and E17.5, likely due to cardiac defects (Supplemental Figure 5). The analysis of *G2-Cre;HIF2dPA* developing hearts at E13.5 revealed thin myocardium, disorganized trabeculae, and regions of the epicardium that stretched away from the underlying myocardium compared with control mice (HIF2dPA mice; Supplemental Figure 5).

**Figure 6:**
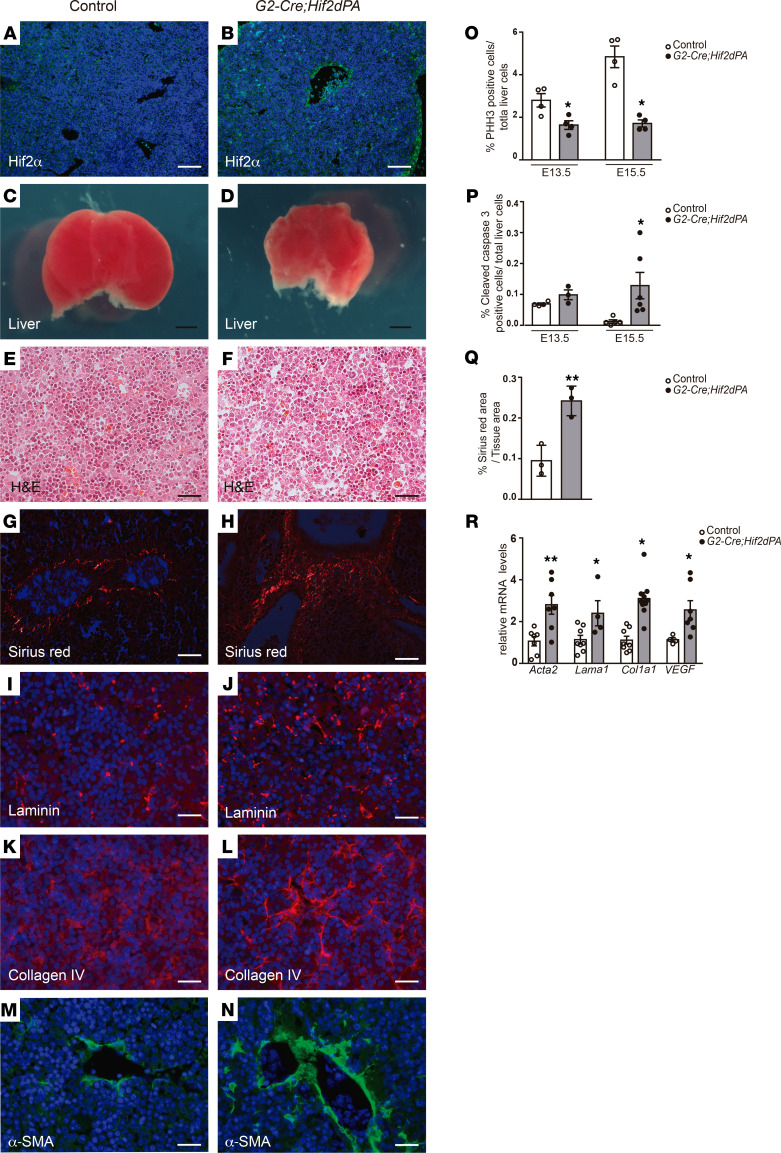
Stabilization of HIF2α protein in HSCs causes liver fibrosis. Immunofluorescence analysis in liver sections of E13.5 (**B**) *G2-Cre;HIF2dPA* embryos demonstrating efficient accumulation of HIF2α compared with (**A**) control embryonic livers. Reduced size of E13.5 (**D**) *G2-Cre;HIF2dPA* embryonic livers compared with (**C**) control livers. (**E** and **F**) H&E-stained sections of E13.5 *G2-Cre;HIF2dPA* and control embryonic livers. (**G** and **H**) Polarized light microscopy images of Sirius red–stained liver sections from E13.5 *G2-Cre;HIF2dPA* and control embryos. Immunofluorescence analysis of (**I** and **J**) laminin, (**K** and **L**) collagen IV, and (**M** and **N**) α-SMA accumulation in liver sections of E13.5 (**J**, **L**, and **N**) *G2-Cre;HIF2dPA* and (**I**, **K**, and **M**) control embryos. Quantification of proliferating liver cells, (**O**) marked by phosphohistone H3 immunoreactivity (*n =* 4 each group), and (**P**) apoptotic liver cells marked by cleaved caspase-3 accumulation (E13.5 *n =* 3–4; E15.5 *n =* 5–6). (**Q**) Quantification of Sirius red–stained area of liver of E13.5 *G2-Cre;HIF2dPA* and control embryos (*n =* 3 each group). (**R**) Quantitative RT-PCR analysis of α*-Sma*, *Lama1*, *Cola1a*, and *VEGF* expression (*n =* 5–8). Scale bars: 100 μm (**A** and **B**); 500 μm (**C** and **D**); 25 μm (**E**–**N**). Statistical analyses was performed using 2-tailed Student’s test. Error bars represent mean ± SEM. *P < 0.05.

A robust accumulation of HIF2α specifically in HSCs of E13.5 *G2-Cre;HIF2dPA* livers was observed by immunofluorescence analysis (Figure 6, A and B). Gross morphological examination of *G2-Cre;HIF2dPA* liver at E13.5 showed reduced size compared with control mice (Figure 6, C and D). The decreased in liver size might be explained by the decrease in cell proliferation as well as increase in cell apoptosis observed in E13.5 and E15.5 *G2-Cre;HIF2dPA* embryonic livers compared with control livers (Figure 6, O and P, and Supplemental Figure 6). Cell proliferation analysis revealed a decrease in both hepatocytes and HSCs of embryonic *G2-Cre;HIF2dPA* livers compared with control livers (Supplemental Figure 6). The histological analyses of E13.5 *G2-Cre;HIF2dPA* livers revealed disrupted architecture with dispersed hepatocytes (Figure 6, E and F). Indeed, a marked increase in laminin and collagen was seen in *G2-Cre;HIF2dPA* embryonic livers (Figure 6, G–L and Q). In agreement with the increase in collagen fibers, a dramatic expansion in the number of α-SMA–positive cells was found in *G2-Cre;HIF2dPA* livers (Figure 6, M and N). Quantification of mRNA levels for ECM and α-SMA confirmed the activation of HSCs and liver fibrosis (Figure 6R). A remarkably increase in *VEGFA* expression, a downstream target of HIF2α, was observed in the livers of *G2-Cre;HIF2dPA* mice (Figure 6R). Altogether, these results indicate that HIF2α activation causes HSCs to become activated leading to a fibrogenic process.

## Discussion

Here, we show that GATA4 is a critical regulator of adult HSC quiescence and, therefore, plays a fundamental role in liver fibrosis. We also found that GATA4 directly regulates *EPAS1* expression. Furthermore, we uncover a possibly previously unknown function of EPAS1 in HSC activation.

We have previously shown that *Gata4* inactivation in embryonic HSCs results in HSC activation. Here, we demonstrate that GATA4 prevents activation of HSCs during adult stages. We have previously generated a Cre line (*G2-Cre*) that targets HSCs very efficiently during embryonic stages (10). However, it also targets epicardial cells and, thus, it might cause extrahepatic defects. To directly test the role of GATA4 in adult HSC activation in vivo we used an adenovirus-mediated strategy. This has been previously proved to be a useful method to genetically manipulate transcription factors in HSCs in vivo (17). In concordance with previous reports, adenovirus infection achieved robust targeting of HSCs (51%) (17). Using this adenovirus-mediated system we show that inactivation of *Gata4* in adult mice caused HSC activation and liver fibrosis. More importantly, we found that adenovirus-mediated *Gata4* overexpression in fibrogenic HSCs induced the regression of liver fibrosis by deactivating aHSCs. This is consistent with *Gata4* expression in HSCs during liver injury and posterior recovery upon cessation of the insult. During injury, *Gata4* expression is lost in aHSCs but, notably, is reexpressed during the HSC deactivation process that takes place through the regression of liver fibrosis. Altogether, our results indicate that reactivation of GATA4 converts aHSC into a quiescent phenotype and, consequently, reverses liver fibrosis.

One potential caveat of the adenovirus approach used in our study is that the observed results might be due to manipulation of GATA4 levels in hepatic cell types other than HSCs. Indeed, hepatocytes are also efficiently targeted by adenoviruses in our experiments. However, even though *Gata4* is highly expressed in hepatoblasts during embryonic development its expression in adult hepatocytes is extremely low or almost negligible (18). Moreover, specific inactivation of *Gata4* in hepatocytes does not result in any apparent defects in liver morphology and function (18). More recently, it has been reported that specific inactivation of *Gata4* in liver sinusoidal endothelial cells (LSECs) using different Cre drivers results in liver fibrosis (19). However, the number of LSECs targeted by adenoviruses in our experiments is very low. Thus, it is unlikely that the liver fibrosis we observed in *Gata4*-floxed mice treated with Ad-Cre might be due to inactivation of *Gata4* in hepatocytes or LSECs rather than HSCs. More importantly, in vitro studies performed with the human HSC LX2 cell line demonstrate that GATA4 can directly revert aHSCs into a quiescent-like, inactivated phenotype.

Our microarray analysis in *Gata4*-overexpressing LX2 cells revealed a large number of differentially regulated genes related to liver fibrosis. *Gata4* overexpression induced downregulation of fibrogenic genes such as ECM components and PDGF and TGF-β receptors (5). Interestingly, we observed increased expression of *TCF21,* a transcription factor that has recently been described to regulate HSC activation (17). Altogether, our data reveal GATA4 as a key component of the transcriptional network regulating adult HSC function. This notion agrees with a very recent report that has identified GATA4 among a group of lineage-specific transcription factors expressed with HSC activation. However, the role of GATA4 in HSC activation was not assessed in vivo in the study (9). Remarkably, the authors found that another member of the GATA family, GATA6, was able to suppress HSC activation in mice. It would be interesting to determine whether GATA4 and GATA6 have complementary roles in HSC biology, as it has shown in other organs.

One of the most interesting genes identified in the gene expression analysis was *EPAS1*, which encodes the hypoxia-inducible factor HIF2α. HIF2α is an oxygen-regulated transcription factor that plays a central role in the cellular response to low levels of oxygen (hypoxia). *Gata4* overexpression decreased *EPAS1* expression in LX2 cells. On the contrary, specific inactivation of GATA4 in HSCs caused an increase in *Epas1* expression in embryonic mouse liver. These results suggested that GATA4 repressed *EPAS1* expression. Indeed, in vitro reporter assays confirmed that GATA4 directly repressed *EPAS1* expression through 2 conserved GATA sites in an intronic region. Although HIFα subprotein levels are regulated mainly by posttranslational mechanisms, transcriptional regulation of *Hif2*α has been reported in different cell types. For example, PGC-1 regulates *Hif*α transcription in muscle fibers and the PI3K/mTORC2 pathway induces transcriptional activation of *Hif2*α in neuroblastoma cells (20). GATA factors can act as activators or repressors in a gene-dependent manner, usually by interacting with other cofactors in specific tissues (21, 22). It would be interesting to determine how GATA4 might interact with other cofactors to regulate the specific transcriptional gene network controlling HSC quiescence.

HIF factors have been associated with several hepatic pathologies, including liver fibrosis. Thus, a profibrogenic effect of HIF1α (another HIFα isoform) has been reported in experimental mouse models of liver injury. In addition, specific activation of both HIF factors (by genetic inactivation of the *Vhl* gene) in hepatocytes causes inflammation and fibrosis in the liver (23). However, these studies have mostly focused on the role of HIF factors in hepatocytes, but their function in HSCs has been barely investigated. Additionally, it has been reported that hypoxia might occur in the liver during chronic injury and that HIF2α promotes liver fibrosis and increases the morbidity of nonalcoholic steatohepatitis via upregulation of NF-κβ pathway (24). Indeed, it has been established that hypoxia promotes the release of factors from HSCs that may affect the progression of fibrosis (25), but the specific contribution of HIF2α has not been analyzed. The expression data in LX2 cells and *Gata4*-deficient mice suggested that increased HIF2α activity might be associated with HSC activation and liver fibrosis. We confirmed this notion in vivo by activating HIF2α specifically in embryonic HSCs using the *G2-Cre* mouse line. We have previously demonstrated that the *G2-Cre* mouse line efficiently targets HSCs during early embryonic development. Stabilization of HIF2α induced activation of HSCs and concomitant liver fibrosis as early as E13.5. How HIF2α activation in HSCs exactly causes liver fibrosis remains to be determined. HIF transcription factors are known to directly regulate a vast number of genes involved in ECM production and vascular remodeling, key biological processes driving organ fibrosis (26). We observed increased expression of VEGF (a well-known HIF2α target) in *G2-Cre;HIF2dPA* embryonic liver and VEGF has been reported to promote liver fibrosis (27). Regardless of the mechanisms involved, our results reveal that HIF2α stabilization might play a role in HSC activation during liver fibrosis. Our results are in concordance with a recent study that has identified EPAS1 as a transcription factor that is upregulated in embryonic HSCs (17). Additional studies in human samples will be necessary to analyze the clinical relevance of HIF2α accumulation in liver fibrosis, including its potential use as a therapeutic target.

Embryos with HIF2α activation directed by *G2-Cre* displayed embryonic lethality. The reasons for this lethality are not entirely clear, but it might be due to cardiac defects. It should be noted that the *G2-Cre* line also targets the epicardium during cardiac development, and aberrant HIFα activity in the developing heart has been shown to cause cardiac defects and embryonic lethality (28).

In summary, our results indicate that GATA4 is an important master regulator of HSC function. In particular, our data indicate that reactivation of GATA4 in aHSC is crucial to induce the regression of liver fibrosis and, thus, GATA4 may represent a potential therapeutic approach for liver fibrosis.

## Methods

### Mice.

*Gata4^fl/fl^*, G2-Cre, ROSA26RYFP, HIF2dPA [Rosa26Sor^Tm4(Hif2A*)Kael^], and G2-Cre mice and strategies for genotyping have been previously described (10, 16, 29, 30).

### Histology, immunohistochemistry, and immunofluorescence.

Dissected livers were fixed in 4% paraformaldehyde in PBS at 4°C overnight and processed for paraffin embedding in a Leica ASP200S tissue processor. Histological analyses and Sirius red staining were performed as described previously (10).

The following primary antibodies were used at the indicated dilutions: rabbit anti–cleaved caspase-3 (1:200, Cell Signaling Technology, 9661); rat anti-CD45 (1:200, BD Pharmingen, BD Biosciences 557390); rabbit anti–collagen IV (1:400, Abcam, Ab19808); goat anti-GFP (1:200, Abcam, Ab6673); mouse anti-GATA4 (1:100, Santa Cruz Biotechnology, SC-25310); goat anti-HNF4α (1:100, Santa Cruz Biotechnology, SC-6556); rabbit anti-laminin (1:50, MilliporeSigma, L9393); mouse anti-HIF2α (1:100, Abcam, Ab8365); rabbit anti-Phospho-Histone H3 (1:500, MilliporeSigma, 06-570); mouse anti-Ki67 (1:100, Thermo Fisher Scientific, RM-9061); mouse anti–α-SMA (1:300, MilliporeSigma, A5228); rabbit anti-desmin (1:100, Abcam, Ab15200); band iotinylated lectin from *Bandeiraea simplicifolia* (L-3759, 1:100, MilliporeSigma). For image quantification, 20 random images at high magnification (40×) of 2 nonconsecutive sections of at least 3 mice per group were quantified using ImageJ software (NIH).

### Microarray analyses.

For microarray analysis, 6 × 10^5^ LX2 cells were cultured in DMEM supplemented with 10% fetal bovine serum in a 10 cm plate and infected with Adenovirus-hGata4 (abm, 093375A) or Adenovirus-GFP (abm, 000541A) at a MOI of 100 over 72 hours. Gene profile was performed in 3 independent experiments for LX2 cells infected with Adenovirus-hGata4 or Adenovirus-GFP using Affimetrix GeneChip Human Gene 2.0ST Array. Differentially expressed genes were defined as those for which the nominal *P* value was 0.05. Gene expression data are available through the Gene Expression Omnibus database (accession number GSE168818).

### Quantitative RT-PCR.

Total RNA from mouse livers or LX2 cells was isolated using the RNeasy Plus Micro kit (Qiagen). cDNA was synthesized using the QuantiTect Reverse Transcription Kit (Qiagen, 205311). Quantitative RT-PCR analysis was performed using a 7500 Real-Time PCR system (Applied Biosystems). RNA expression of target genes was normalized based on comparison to the housekeeping gene. The ΔΔCt method was used to calculate changes in gene expression levels. Quantitative qPCR was performed with at least 5 independent samples in triplicate. qPCR was performed using the following TaqMan probes (Applied Biosystems): ACTA2 Hs00426835_g1, COL1A1 Hs00164004_m1, LAMA1 Hs00300550_m1, EPAS1 Hs0102649_m1, TLR4 Hs00152939_m1, IL-6 Hs00174131_m1, TIMP1 Hs01092512_g1, βACT Hs03023880_g1, GATA4 Mm00484689_m1, LAMA1 Mm01226102_m1, COL1A1 Mm00801666_g1, EPAS1 Mm01236112_m1, ACTA2 Mm00725412_s1, VEGF Mm00437306_m1, TGF-β Mm01178820_m1, and βACT Mm02619580_g1 (Applied Biosystems). The primers for Sybr green qPCR are listed in Supplemental Table 1.

### Epas1 silencing by RNA interference.

Specific siRNA for human *Epas1* (HSS103261) and Stealth siRNA negative control (12935300) were purchased from Invitrogen. For transfection, 30 pmol of the corresponding siRNA were used for transfecting 2 × 10^5^ LX2 cells cultured in 6-well plates using Lipofectamine 2000 (Invitrogen). After 72 hours of incubation, cells were collected for RNA isolation and qPCR analyses.

### ChIP.

ChIP assays were performed using Dynabeads Protein A (100.01D, Invitrogen) following the manufacturer’s recommendations, with some modifications. LX2 cells were grown in DMEM supplemented with 10% FBS on a 15 cm plate to approximately 7 × 10^6^ cells and were infected with Ad-GATA4 at a MOI of 100. After 72 hours, cells were treated with 1% formaldehyde for 30 minutes to crosslink protein-DNA complexes. Cells were lysed and sonicated to shear the DNA. The cleared supernatant was divided into 2 samples. One of the samples was incubated with 5 μg anti-GATA4 antibody (Santa Cruz Biotechnology, SC-25310) and the other sample was incubated with 5 μg IgG (Santa Cruz Biotechnology) as a nonspecific antibody for 2 hours at room temperature. Following incubation in 5 M NaCl at 65°C overnight to reverse the crosslinks, DNA was recovered by phenol-chloroform extraction. For Chip-qPCR of *EPAS1* the following primers were used: 5′-GTTGCTGGACACACCACATTTC-3′ and 5′-CCCTAAGTCCCGGGTACAG-3′. Percentage input method was used to calculate enrichment of GATA4 binding to *EPAS1* DNA.

### CCl4-induced liver fibrosis and adenovirus treatment.

Three- to four-month-old female and male C57Bl/6 mice were treated with intraperitoneal injections of 0.5 mL/kg of body weight CCl_4_ (319961-1L, MilliporeSigma) dissolved in olive oil (1:1) (O1514, 500 mL, MilliporeSigma) twice per week for 4 weeks (8 injections total) to induce liver fibrosis. For adenovirus treatment, mice were injected via tail vein with 1.8 × 10^9^ infection unit (IFU) of Ad-GATA4 or Ad-GFP as a control 48 hours after the last CCl_4_ injection. Mice were sacrificed for analysis 1 week after the adenoviral injection.

For *Gata4* inactivation in adult liver, *Gata4*-floxed mice were injected via the tail vein with 4.6 × 10^8^ IFU Ad-Cre (SignaGen Laboratories, SL100707) or Ad-GFP as control. Mice were sacrificed for analyses 2 weeks after adenoviruses treatment.

### Cloning, cell culture, transfection experiments, and reporter assays.

An 810 pb fragment of the human *EPAS1* gene was generated by PCR using the following 2 primers: forward 5′-AATCAGGATGGTACCTAGAAGCATAT-3′ and reverse 5′-GCCTAAAACAGCTCGAGGATCAGCACC-3′. This fragment was then cloned into pGL3 reporter vector (Promega) containing the thymidine kinase minimal promoter to generate the pGL3-*EPAS1* *wt* vector. Mutations of the GATA sites in the EPAS1 fragment were introduced by PCR using the following primers: forward 5′-TTACCCAGATCTTAAGCTTAGGAAAG-3′ and reverse 5′-CTTTCCTAAGCTTAAGATCTGGGTAA-3′. The *EPAS1* fragment containing the mutated GATA sites was cloned into pGL3 reporter vector to generate the pGL3-*EPAS1* *mut*. 293T cells were maintained in DMEM supplemented with 10% FBS. Cells were cultured in 6-well plates at a density of 2 × 10^5^ cells per well and transfected using Lipofectamine 2000 (Promega) following the manufacturer’s recommendations. Each well was transfected with 2.5 μg luciferase reporter plasmids. The firefly luciferase activity was normalized to renilla luciferase activity by cotransfecting each well with 0.5 μg pRL-renilla reporter vector (Promega, E1910). Cells were collected 72 hours after transfection, and the luciferase activity was measured using the Dual Luciferase Kit Assay (Promega) and detected in a luminometer (Glomax 20/20, Promega).

### Western blot.

Cells and liver samples were lysed and homogenized in Protease Inhibitor buffer (S8820, MilliporeSigma). Protein concentration was determined using Quick Start Bradford 1X Dye Reagent (5000205, Bio-Rad). Electrophoretically separated proteins were transferred to PVDF membranes (IPFL00010, MilliporeSigma) and incubated with the following primary antibodies: mouse anti–α-SMA (1:1000, MilliporeSigma, A5228), rabbit anti–collagen IV (1:1000, Abcam, Ab227616), mouse anti-GATA4 (1:1000, R&D Systems, MAB2606), rabbit anti-SMAD7 (1:100, Invitrogen, 42-0400), mouse anti-phosphoSTAT1 (Tyr701) (1:500, Invitrogen, 33-3400), rabbit anti-STAT1 (1:1000, Cell Signaling, 9172), rabbit anti-GAPDH (1:15,000, Cell Signaling, 2128), and mouse anti–β-actin (1:10,000, MilliporeSigma, A5441). Secondary antibodies rabbit anti-IRDye 800CW (LI-COR, 925-32211), mouse anti-IRDye 680RD (925-68070), mouse anti-IgG-peroxidase (MilliporeSigma A9044), and rabbit anti-IgG peroxidase (MilliporeSigma A0545) were used. The Odyssey CLx Infrared Imaging System (LICOR) or enhanced chemiluminescence detection reagents (GE Healthcare Bio-Sciences Corp.) in the Image Quant 800 System (Amersham) was used for imaging the blots.

### Statistics.

Statistical analyses were performed using GraphPad Prism (version 7.0) and the 2-tailed Student’s test or 2-way ANOVA. *P* < 0.05 was considered statistically significant.

### Study approval.

All experiments using animals complied with institutional guidelines RD 53/2013 and the EU Directive 2010/63/EU and were reviewed and approved by the Institutional Animal Care and Use Committee of the University of Seville (Seville, Spain).

## Author contributions

LV, NA, ID, RC, MRR, and MGT performed the experiments. LP, RMC, FM, DAC, and AR procured and analyzed the data. DAC and AR wrote the manuscript. All authors edited the manuscript. AR is the guarantor of this work and, as such, had full access to all the data in the study and takes responsibility for the integrity of the data and the accuracy of the data analysis.

## Supplementary Material

Supplemental data

## Version 1 10/26/2021

In-Press Preview

## Version 2 12/08/2021

Electronic publication
