# Supplementary material for: GATA4 induces liver fibrosis regression by deactivating hepatic stellate cells
Source: JCI Insight. 2021 Dec 8;6(23):e150059. doi: 10.1172/jci.insight.150059 (PMC8675192; doi:10.1172/jci.insight.150059)

Supplemental Figure 1. Adenovirus-mediated targeting of HSCs (A-C) Immunofluorescence analysis of liver sections of adult C57BL/6 wild type mice injected with GFP-expressing adenoviruses (Ad-GFP). Pictures of liver cells co-expressing GFP and the HSCs marker desmin (A), the hepatocyte marker HNF4 (B) and Lectin from *Bandeiraea simplicifolia* (C). Arrowheads indicate cells co-expressing both markers. (D) Quantification of double labelled cells for GFP and Desmin, GFP and HNF4, and GFP and lectin (percentage per total cells) in liver of adult mice C57BL/6 wild type injected with Ad-GFP (n=3 each group). (E) PCR using specific primers for *Gata4* on genomic DNA from liver of wild type (+/+), *Gata4* floxed mice injected with *Cre*-expressing adenovirus (*Gata4* $^{flox/flox}$; Ad-Cre) or GFP-expressing adenovirus (*Gata4* $^{flox/flox}$; Ad-GFP) demonstrates Cre-mediated excision of the *Gata4* floxed alleles. Polarized light microscopy pictures of Sirius-red-stained liver sections of Gata4$^{+/+}$ control mice injected with *Cre*-expressing adenovirus (Ad-Cre) (F) or with GFP-expressing adenovirus (Ad-GFP) (G) and *Gata4*$^{flox/flox}$ mice injected with GFP-expressing adenovirus (Ad-GFP) (H). (I) Quantification of Sirius red-stained area of liver per total tissue area in each experimental group (n=3 each group). Scale bars= 25µm for A-B; 100µm for C, F-H. Statistical analyses was performed using two-tailed Student Test. Error bars represent mean ± SEM.

Supplemental Figure 2. Labelling of *G2Cre*-targeted cells. (A) Immunofluorescence analysis of YFP and Desmin accumulation in liver sections of adult *G2-Cre; ROSA26ReYFP* mice. (B) Quantification of cells coexpressing YFP and Desmin relative to total Desmin-positive cells in liver of *G2-Cre; ROSA26ReYFP* mice (n=3). Scale bars: 25µm.

Supplemental Figure 3. CCl_4-treatment induce liver fibrosis similarly in male and female mice. (A) Relative quantification of Sirius red-stained area per total liver area in

CCl_4-treated and control-treated (oil) adult male and female C57BL/6 mice. Polarized light microscopy pictures of Sirius-red-stained liver sections from CCl_4-treated male (D) and female mice (E) and control-treated (oil) (B) male and female mice (C) (n=3). (F) Serum AST levels in CCl_4-treated and control-treated (oil) male and female adult mice (n=3 oil; n=6 CCl_4). (G) Serum ALT levels in CCl_4-treated and control-treated (oil) male and female adult mice (n=3 oil; n=6 CCl_4). Scale bars: 100µm. Statistical analyses was performed using two-tailed Student Test. Error bars represent mean ± SEM. **p < 0.01.

Supplemental Figure 4. *Gata4* expression during liver fibrosis regression. (A) Quantitative RT-PCR analysis of *Gata4* expression and relative quantification of Sirius red-stained area per total liver area (B) (n=3-4) of mice treated with vehicle (oil), CCl_4 and two-three and 4-weeks after CCL_4 treatment (recovery phase) (n=3-4). Statistical analyses was performed using one-way ANOVA test. Error bars represent mean ± SEM. *p < 0.05, **p < 0.01, **p < 0.001.

Supplemental Figure 5. Vascular hemorrhage in *G2-Cre;HIF2dPA* embryos. Representative pictures of whole E13.5 *G2-Cre;HIF2dPA* (B) and control littermate embryos (A). (C, D) Immunostaining for HIF2α in E13.5 embryonic hearts of control and *G2-Cre;HIF2dPA* embryos. Note the accumulation of HIF2α protein in the epicardium of *G2-Cre;HIF2dPA* embryos (arrows in D) compared with control embryos. Hematoxylin-eosin staining of heart sections of E13.5 *G2-Cre;HIF2dPA* (F) and littermate control embryos (E). (G, H) Higher magnification images of panels E and F. The hearts of E13.5 *G2-Cre;HIF2dPA* embryos display a thinner ventricular septum and a more compacted myocardium (marked by double arrows) compared to control embryonic hearts. The hearts of E13.5 *G2-Cre;HIF2dPA* embryos show lack of

myocardial compaction, ventricular trabeculae close to the epicardium (J, arrowheads) and epicardium distended from the underlying myocardium (J, arrows) compared to control hearts (I). At least 4 embryos per experimental group (from two independent litters) were analyzed. Scale bars: 500µm for A, B, E and F; 100µm for C, D, G and H; 25µm for I and J.

Supplemental Figure 6. Proliferation analysis in *G2-Cre;HIF2dPA* liver embryos. Hepatocytes (marked by HNF4α immunoreactivity) of E15.5 embryonic *G2-Cre;HIF2dPA* livers (B) show decreased proliferation (assessed by Ki-67 immunofluorescence) compared with control embryonic livers (A). Arrowheads in A and B indicate double labelled cells for HNF4α and Ki-67. (C) Quantification of proliferating hepatocytes (positive for HNF4α) in E15.5 embryonic livers (n=3 each group). Reduced hepatic stellate cell (marked by desmin immunofluorescence) proliferation in E15.5 embryonic *G2-Cre;HIF2dPA* livers (E) compared with control embryonic livers (D) (n=3 each group). Arrows in D and E indicate double labelled cells for Desmin and Ki-67. (F) Quantification of proliferating hepatic stellate cells (positive por desmin) in E15.5 embryonic livers (n=3 each group). Immunostaining for cleaved Caspase 3 in control liver (G) or *G2-Cre;HIF2dPA* (H) of E15.5 embryos. Scale bars: (A, B, D, E, G, H): 10µm. Statistical analyses was performed using two-tailed Student Test. Error bars represent mean ± SEM. *$p < 0.05$.

Supplemental Table 1: List of primers used for RT-PCR

GENE	SEQUENCES
mouse *Gata4* deletion floxed alleles	Rec2: TCCATGAGACCCCAGAGTGTGCCTGAG<br>Rec4: ACCCTGGAAGACACCCCAATCTCGG<br>Rec5: TGTCATTCTTCGCTGGAGCCGC
mouse TIMP1	Forward ACC TGG TCA TAA GGG CTA AA<br>Reverse ATT TCC CAC AGC CTT GAA TC
mouse IL-6	Forward CAG AGT CCT TCA GAG AGA TAC A<br>Reverse GTG ACT CCA GCT TAT CTG TTA G
mouse TGFBR1	Forward TGC CAT AAC CGC ACT GTC A<br>Reverse AAT GAA AGG GCG ATC TAG TGA TG
mouse TGFBR2	Forward GAG TCG TTC AAG CAG ACG GA<br>Reverse GAA CCA AAT GGG GGC TCG TA
mouse PDGFRA	Forward GGA AGG CAC AGA AGC AAT A<br>Reverse GGC TCA GTC TTC ACA CTT AC
mouse PDGFRB	Forward GTC CTT ACC GTC ATC TCT CT<br>Reverse CAC AGA CTC AAT GAC CTT CC
mouse TLR4	Forward CAA CAT CAT CCA GGA AGG C<br>Reverse GAA GGC GAT ACA ATT CCA CC
mouse SMAD7	Forward GGG CTT TCA GAT TCC CAA CTT<br>Reverse CAC GCG AGT CTT CTC CTC C
mouse STAT1	Forward GAT CTC TAA CGT CTG TCA GCT G<br>Reverse GAG GTC CAG GAT TCC TTC GA TC
mouse TCF21	Forward AGG TCA TTC TCT GGT TTG CC<br>Reverse GCT ACA TCG CTC ACT TAA GGC
mouse GATA4 EX2	Forward GTG GCC CTG GCG CCT TCA TG<br>Reverse TCC CAG GCC CTG CAC CCG AC
mouse β-ACT	Forward TCC TGT GGC ATC CAC GAA ACT<br>Reverse ACC AGA CAG CAC TGT GTT GGC
human TGFBRA1	Forward GGA CCA TTG TGT TAC AAG A<br>Reverse CCA TGC TCA TGA TAA TCT G
human TGFBRA2	Forward GTA GCT CTG ATG AGT GCA A<br>Reverse CAG ATA TGG CAA CTC CCA G
human PDGFRA	Forward AAA GAA GTT CCA GAC CAT C<br>Reverse AGG TGA CCA CAA TCG TTT CC
human PDGFRB	Forward CAG CAA GGA CAC CAT GCG G<br>Reverse GGG GCT CCT GGG ACA TCC GT
human SMAD7	Forward AGA AGG TGC GGA GCA AAA T<br>Reverse GTG TGG CGG ACT TGA TGA
human STAT1	Forward CTA GTG GAG TGG AAG CGG A<br>Reverse CAC CAC AAA CGA GCT CTG AA
human TCF21	Forward TCC TGG CTA ACG ACA AAT AC<br>Reverse TTT CCC GGC CAC CAT AAA GG
human β-ACT	Forward GAT CAG CAA GCA GGA GTA TG<br>Reverse AAG GGT GTA ACG CAA CTA AG

Supplemental Figure 1

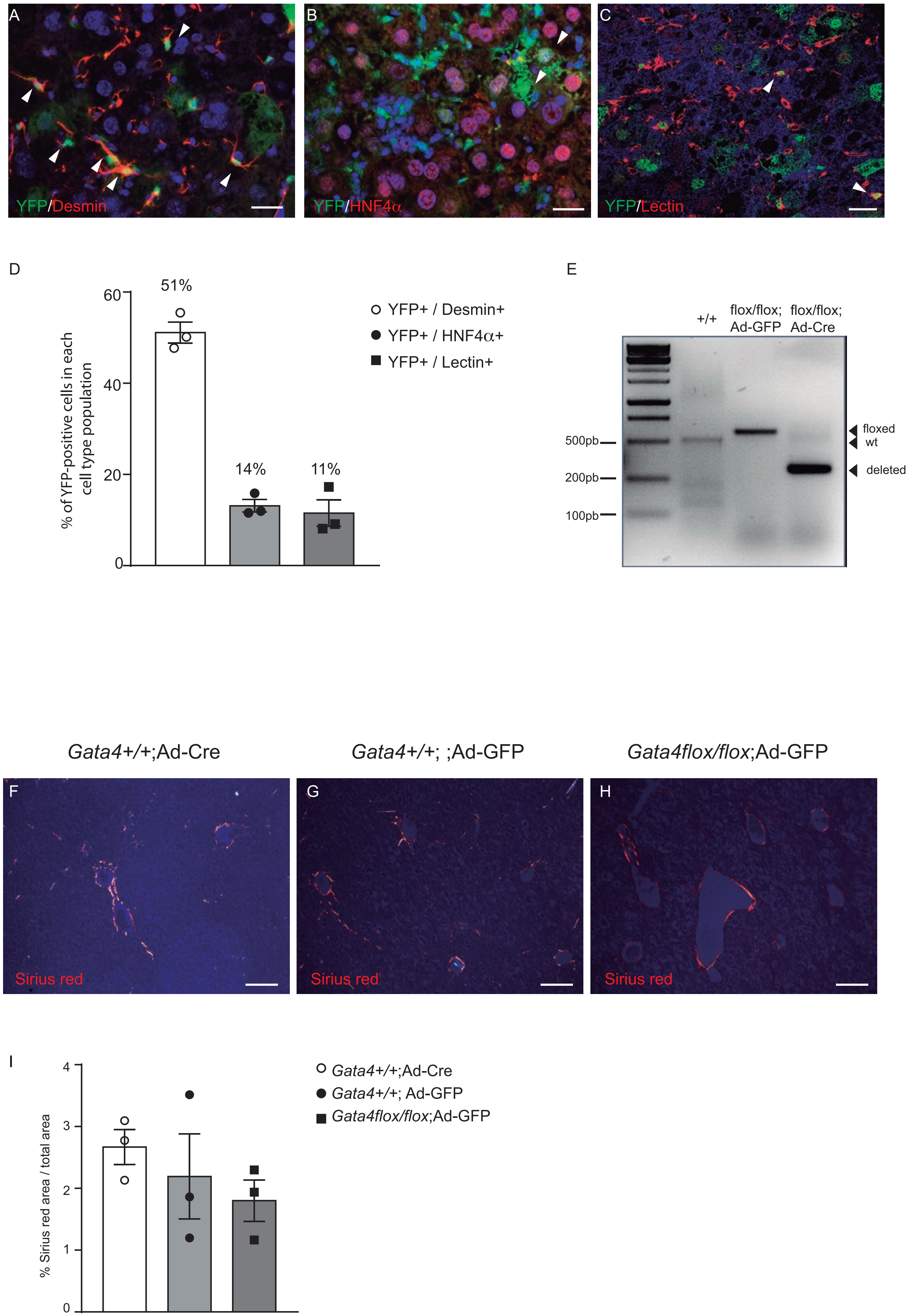

Supplemental Figure 2

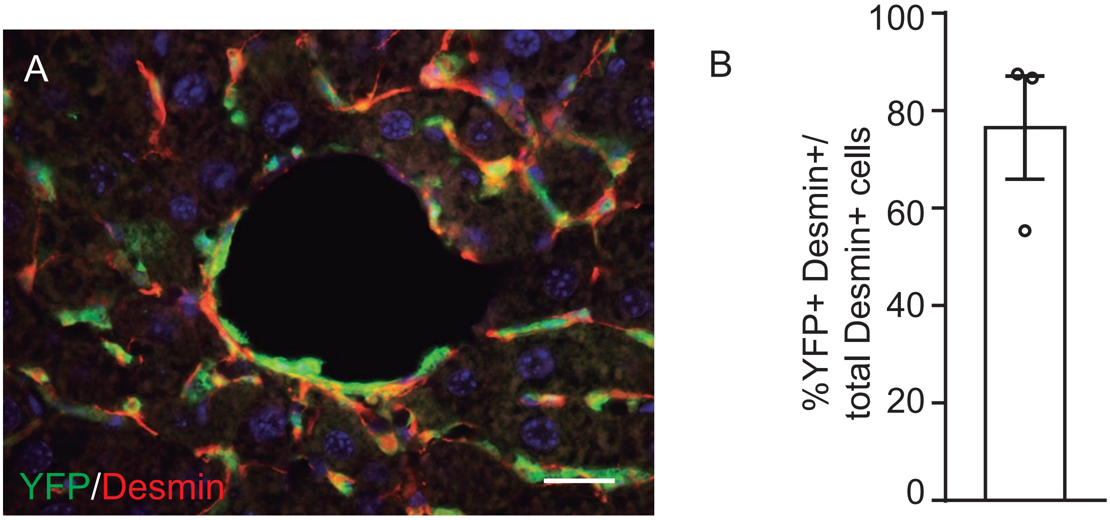

Supplemental Figure 3

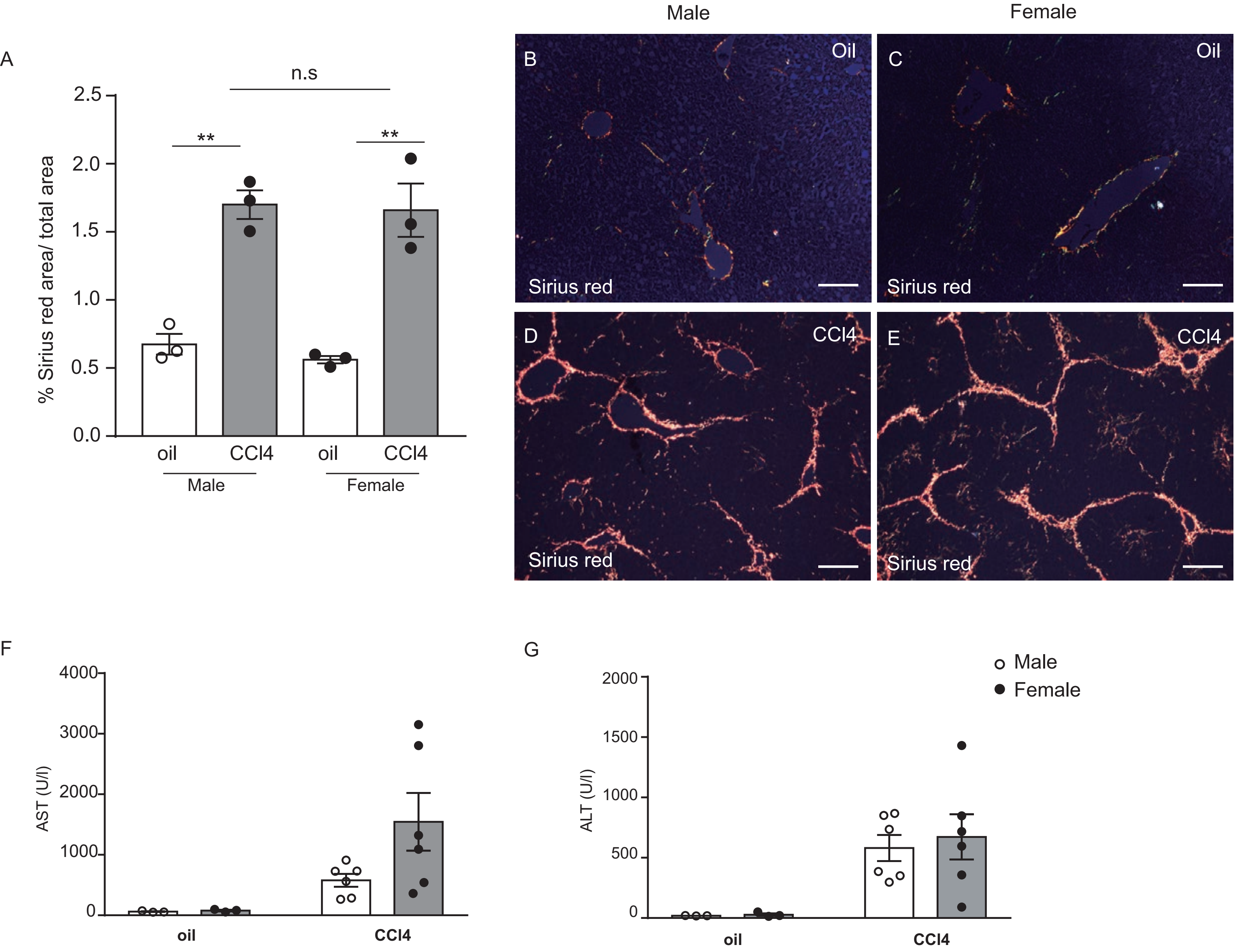

Supplemental Figure 4

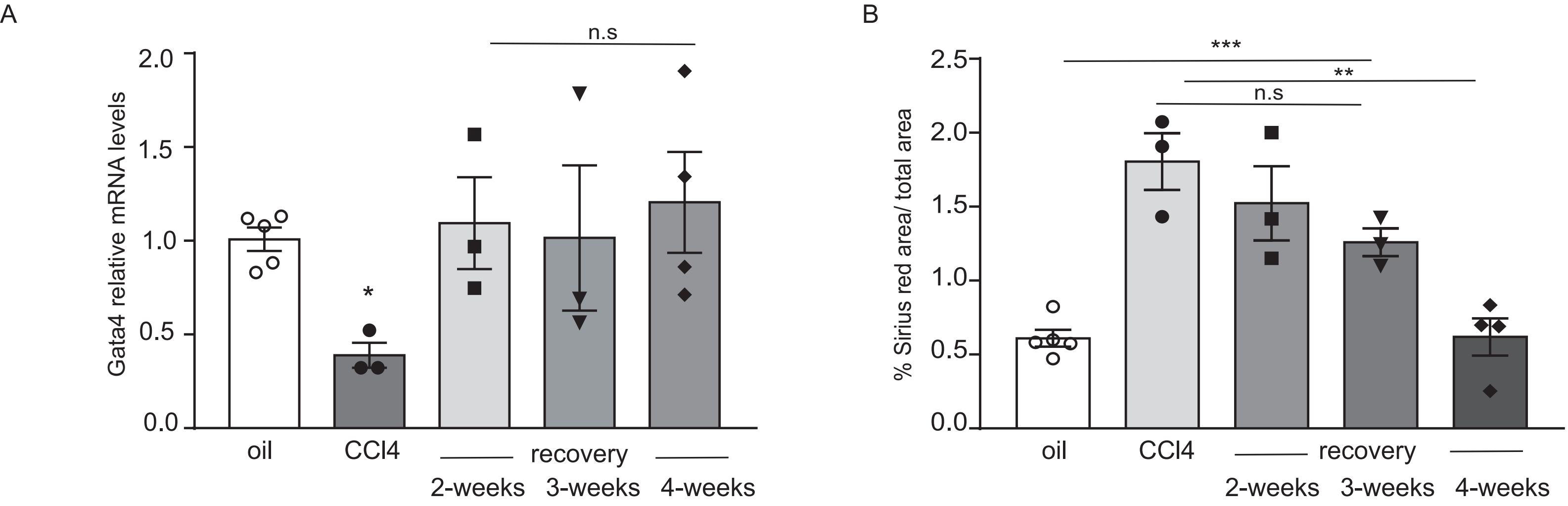

Supplemental Figure 5

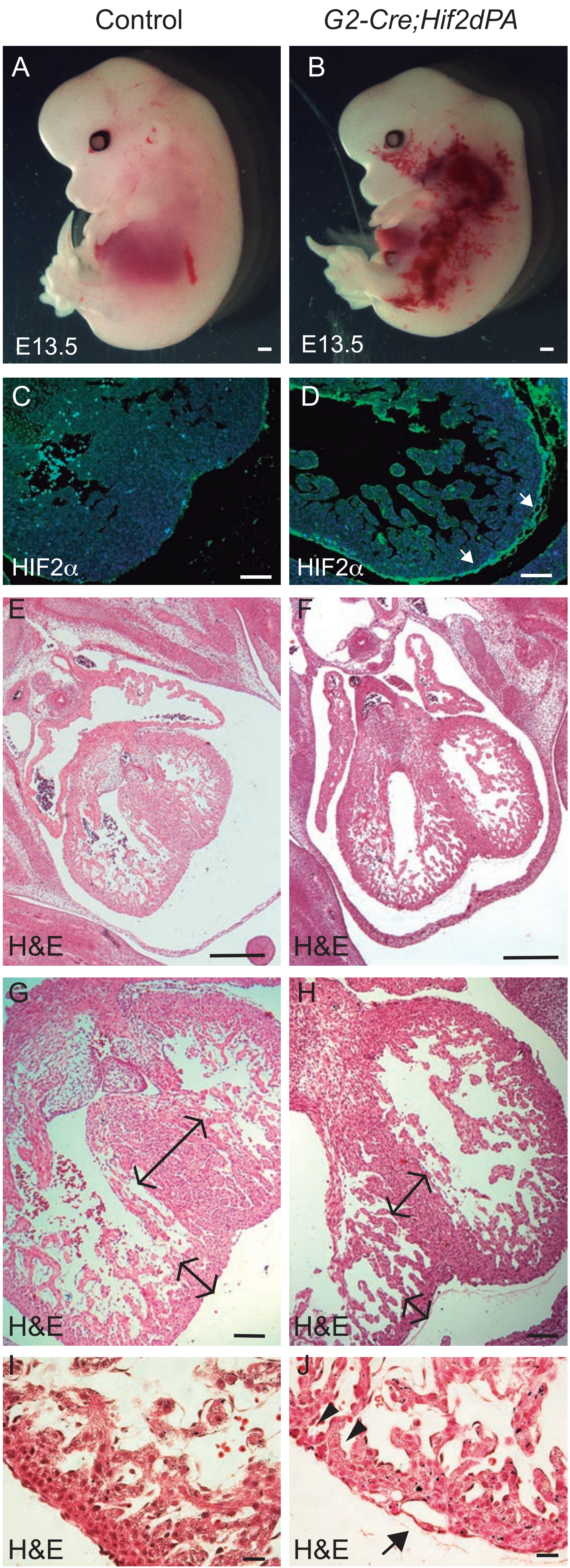

Supplemental Figure 6

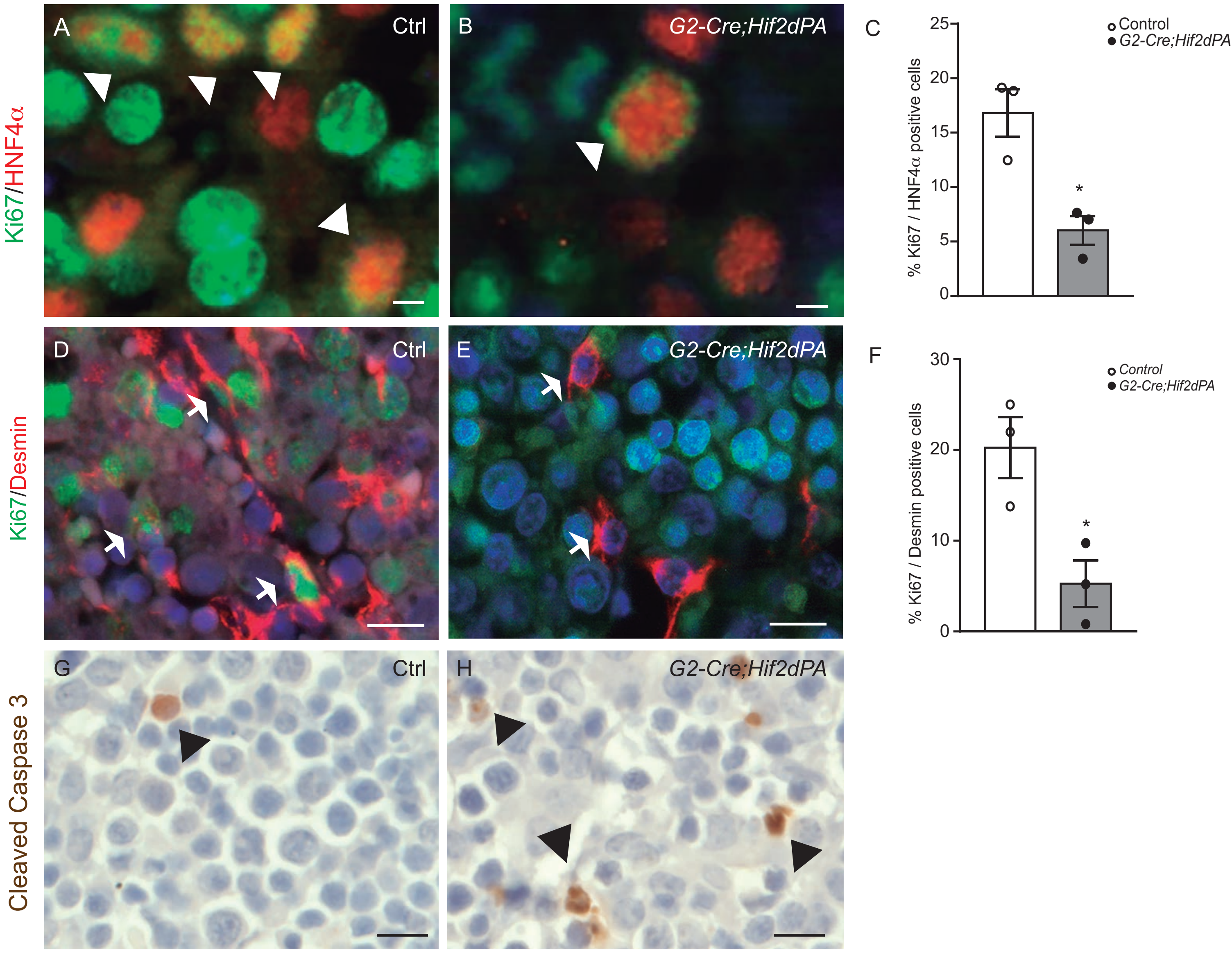

Uncropped gels Figure 2

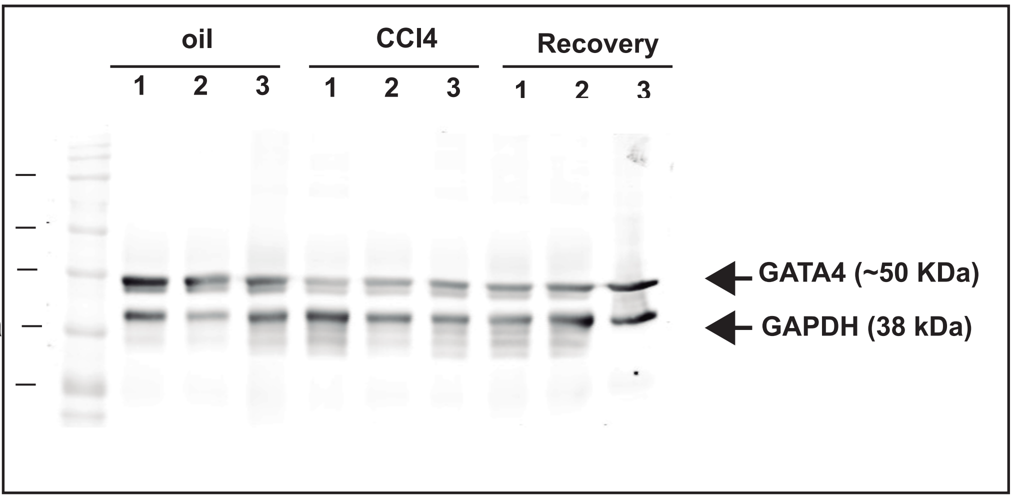

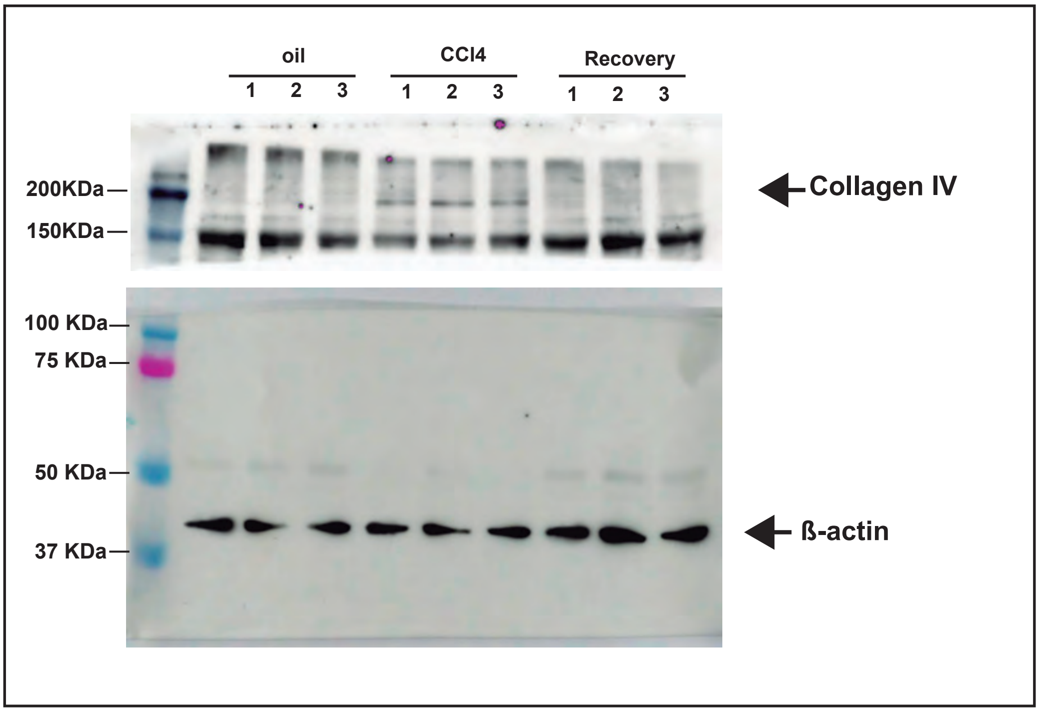

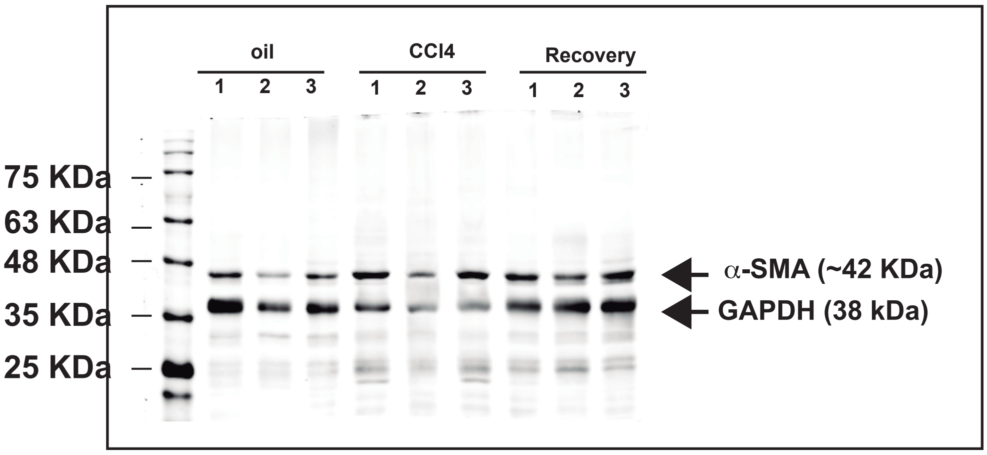

Uncropped gels Figure 4

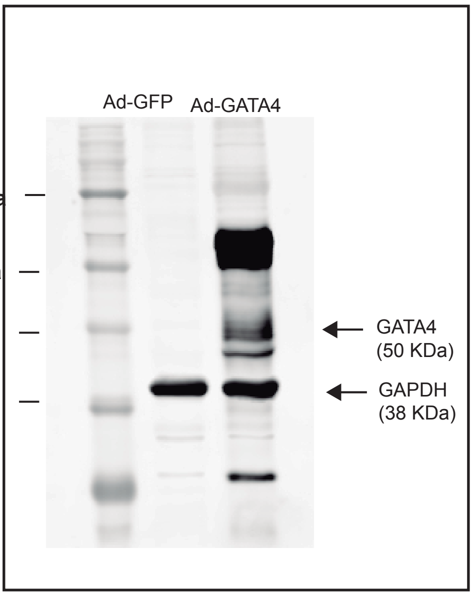

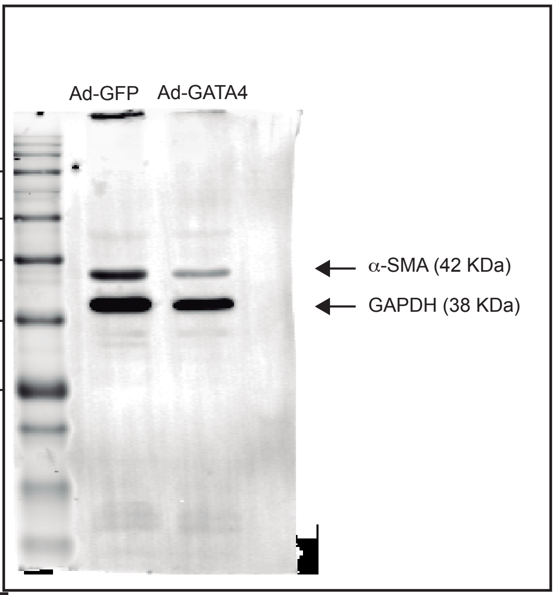

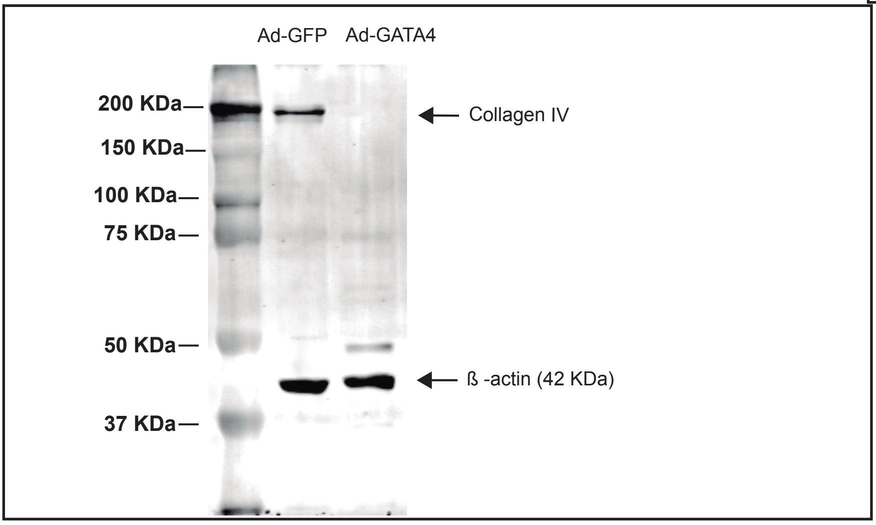

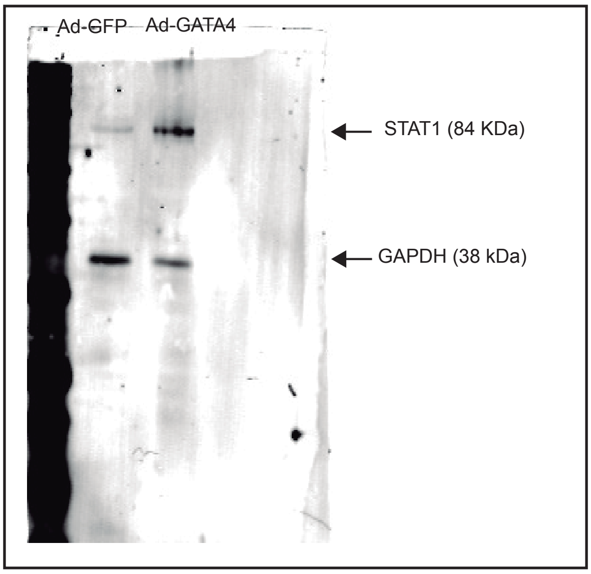

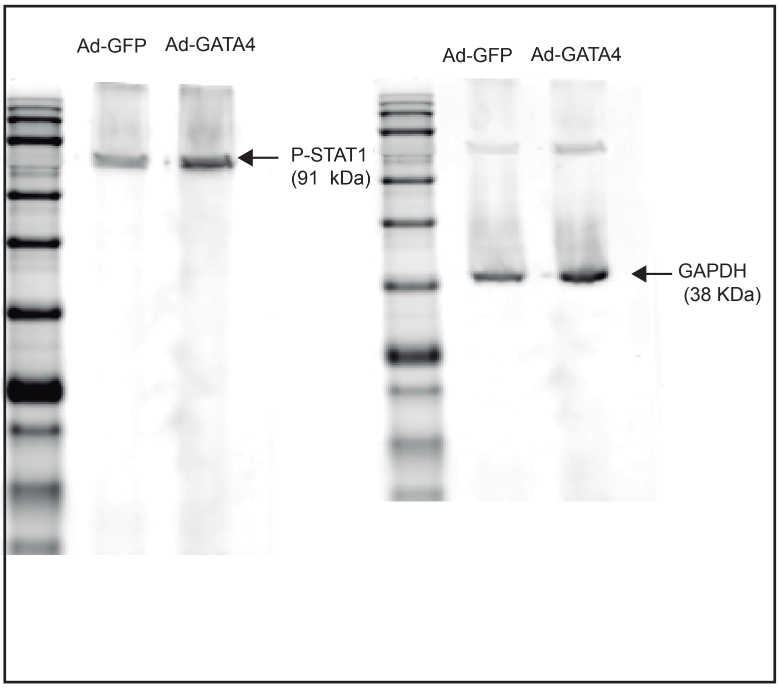

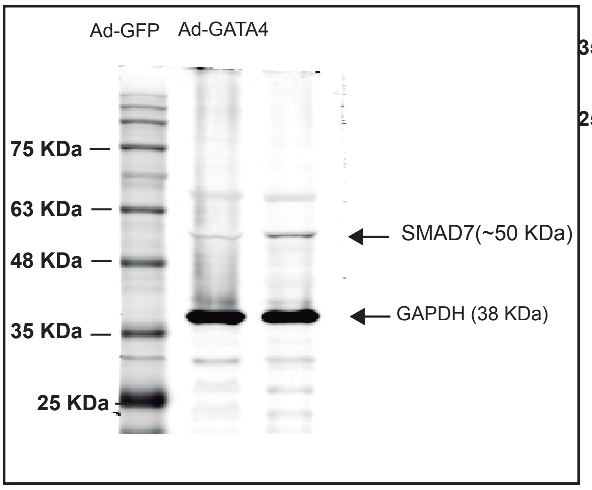

Supplement: Supplemental data [file jciinsight-6-150059-s058.pdf]
